# Role of LFA-1 integrin in the control of a lymphocytic choriomeningitis virus (LCMV) infection

**DOI:** 10.1080/21505594.2020.1845506

**Published:** 2020-11-29

**Authors:** Mario Perro, Matteo Iannacone, Ulrich H. von Andrian, Antonio Peixoto

**Affiliations:** Harvard Medical School, Department of Microbiology and Immunobiology, Boston, Massachusetts, USA

**Keywords:** Integrin, T cell activation, LCMV, T cell differentiation

## Abstract

Leukocyte function-associated antigen 1 (LFA-1) is the most widely expressed member of the β2 integrin family of cell-cell adhesion molecules. Although LFA-1 is thought to regulate multiple aspects of T cell immunity, its role in the response of CD8^+^ T cells to viral infections remains unclear. Indeed, compelling clinical evidence shows that loss of LFA-1 function predisposes to infection in humans but animal models show limited to no susceptibility to infection. Here, we addressed this conundrum in a mouse model of infection with lymphocytic choriomeningitis virus (LCMV), where CD8^+^ T cells are necessary and sufficient to confer protection. To this end, we followed the fate and function of wild-type and LFA-1 deficient virus-specific CD8^+^ T cells and assessed the effect of blocking anti-LFA-1 monoclonal antibody in the outcome of infection. Our analysis of viral clearance and T cell responses using transcriptome profiling reveals a role for LFA-1 as a gatekeeper of effector T cell survival and dysfunction that when defective can predispose to LCMV infection.

## Introduction

The leukocyte adhesion molecule LFA-1 and its ligands have been implicated in several aspects of T cell biology, including the migration of both naïve (T_N_) and effector (T_EFF_) T cells from blood into tissues [1]. For T_N_, LFA-1 mediated adhesion to high endothelial venules is critical for access to lymph nodes (LN) where T_N_ encounter antigen-presenting cell (APCs) and receive survival signals, such as interleukin-7 [2]. When T_N_ interact with cognate APCs they become activated, leading to their retention, proliferation, and differentiation within the LN. The activated cells ultimately give rise to large numbers of effector cells (T_EFF_) that can respond to re-encounter of their specific antigen by secreting cytokines, such as interleukin-2 (IL-2), interferon-γ (IFNγ) or tumor necrosis factor-α (TNFα), and/or by killing APCs. After exiting the LN, T_EFF_ return to the blood stream and migrate to infected peripheral tissues to eliminate infected cells [3]. Once the pathogen has been cleared, most T_EFF_ undergo apoptosis, but a small subset of Ag-experienced T cells persists as long-lived memory cells (T_MEM_), which are further subdivided into tissue-resident (T_RM_) as well as recirculating central and effector memory subsets (T_CM_ or T_EM_, respectively). T_N_ do not require LFA-1 to migrate within an LN [4], but LFA-1 is required for persistent contacts with APCs [5]. Furthermore, LFA-1 transmits inside-out and outside-in signals [6] and modulates T cell receptor (TCR) signaling by participating in the formation of the immunological synapse [7]. At later stages of the immune response T_EFF_ often require LFA-1 to migrate to peripheral sites of infection and to form a lytic synapse with infected target cells [8].

Consequently, leukocyte adhesion deficiency syndrome type 1 (LAD1), a human genetic deficiency in the β2 integrin chain (the shared subunit of the β2 integrin family, which includes LFA-1), predisposes to infection in humans [9]. Moreover, blocking LFA-1 with a humanized monoclonal antibody (MAb) predisposes patients to progressive multifocal leukoencephalopathy (PML), an often fatal CNS infection caused by JC virus [10]. LFA-1 deficient (LFA-1^−/-^) mice also display multiple abnormalities, such as deficient T cell homing [11], reduced numbers of regulatory T cells [12] and compromised NK cell activity [13]. Consequently, LFA-1^−/-^ mice are more susceptible to pulmonary infection with *Streptococcus pneumoniae* and *Mycobacterium tuberculosis* [14,15]. However, LFA-1 deficiency predisposes only against some, but not all pathogens. For example, LFA-1^−/-^ mice are equally susceptible as wild-type (WT) mice to infection by lymphocytic choriomeningitis virus (LCMV) serotype Armstrong (LCMV-ARM) [13], and even protected against intravenous infection with *Listeria monocytogenes* [16].

Despite years of study, LFA-1’s precise contribution to T cell priming, T_EFF_ differentiation/function remains incompletely characterized. Moreover, the differential requirements of LFA-1 to fight some pathogens and not others remain elusive. To shed light on these matters, we employed a mouse model of infection with two strains of LCMV, LCMV-ARM, and LCMV clone 13 (LCMV-CL13). In this model, a low dose challenge (5x10^4^pfu) with either strain induces a CD8^+^ T cell response resulting in clearance of the infection that is strictly dependent on CD8^+^ T cells [17]. The genomes of these strains differ in five-point mutations resulting in two amino-acid changes that confer higher replication capacity and infectivity to the LCMV-CL13 strain [18,19]. Therefore, in WT mice challenged with a high dose (2x10^6^pfu) of LCMV-CL13 (but not LCMV-ARM), anti-viral CD8 T cells become functionally exhausted, leading to chronic infection [20].

Our work shows that upon a low dose challenge with LCMV (5x10^4^pfu), loss of functional LFA-1 reduced the burst size of the anti-viral CD8 T_EFF_ response against both LCMV ARM and CL13 strains. The reduced CTL burst size was related to the upregulation by T_EFF_ of several cell death pathways that involved TNF, Fas and caspases. However, while the loss of LFA-1 function had little impact on the course of infection with LCMV-ARM, it prevented the control of LCMV-CL13 infection. Indeed, when LFA-1 was inhibited in LCMV-CL13 infected mice, virus-specific CD8 T_EFF_ displayed functional and transcriptional characteristics that were reminiscent of T cell exhaustion and tolerance dysfunctional states. Surprisingly, we observed a transcriptomic signature of low-grade exhaustion on CD8 T_EFF_ in the absence of LFA-1 during an acute infection with LCMV-CL13. Hence, CD8^+^ T cell activation in the absence of LFA-1 leads to a CTL dysfunctional state that can be exacerbated by prolonged exposure to viremia, thus preventing the control of a high replicating virus such as LCMV-CL13. In summary, we provide new and important insights into a mechanism governing antiviral CD8^+^ T cell function.

## Results

### Effect of anti-LFA-1 MAb treatment on anti-viral T_EFF_ burst size

The capacity of mice to control an LCMV infection varies with both the dose and virulence of the infecting strain [19]; thus, we asked whether the requirement for LFA-1 depends on these factors. To this end, we challenged C57BL/6 mice with LCMV-ARM or LCMV-CL13 while the animals were treated for 7 days with a non-depleting anti-LFA-1 MAb that blocks LFA-1 dependent T cell adhesion [2]. This strategy does not induce cell depletion and saturates the vast majority of LFA-1 molecules on the surface of CD8 T cells (**Fig. S1**). Furthermore, it avoids potential defects in anti-viral immunity due to aberrant T cell development in LFA-1 deficient mice [21,22]. The viral dose (5x10^4^ pfu) was chosen to enable untreated C57BL/6 mice to control the infection by either strain of LCMV.

**Figure 1.**
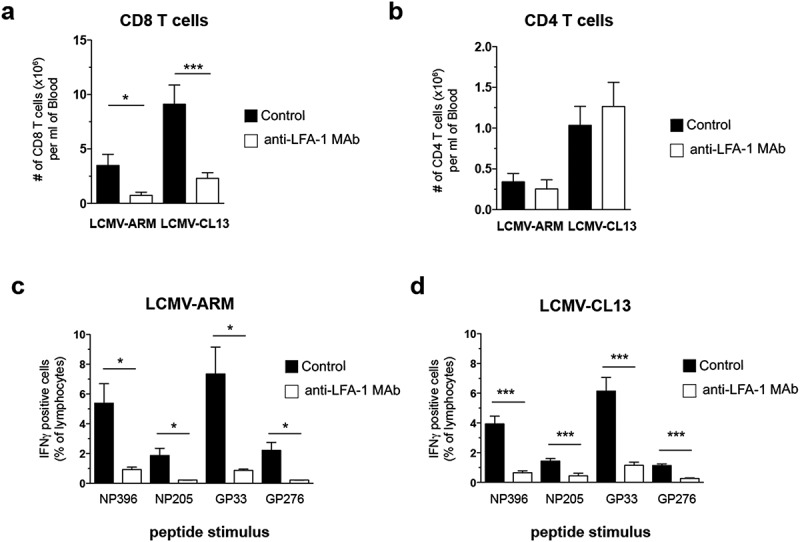
Effect of anti-LFA-1 Mab treatment on anti-viral T_EFF_ burst size

Our results show that, in animals that had received anti-LFA-1 Mab during infection with LCMV-ARM or LCMV-CL13, the number of circulating CD8 T cell was ~4-fold decreased when compared to infected control mice (Figure 1(a)). In contrast, circulating CD4 T cells were not affected, suggesting that LFA-1 is dispensable for the burst size of CD4 T cells (Figure 1(b)). In addition, we observed a profound reduction in the LCMV-specific CD8 T_EFF_ response in anti-LFA-1 treated mice that was similar for several LCMV epitopes, regardless of the viral strain used for challenge (Figure 1(c, d)).

Total numbers of CD8 (a) and CD4 (b) T cells assessed in the blood of LCMV-infected mice at day 9 post-infection (p.i.). (c,d) Frequency of immunodominant (NP396, GP33) and subdominant (NP205, GP206) LCMV-specific CD8+ T_EFF_ in the blood of infected mice at day 9 p.i. detected by *ex vivo* re-stimulation with different LCMV peptides. See also Figure S1.* p < 0.05, ** p < 0.001, *** p < 0.0001. Error bars represent mean±SEM. Three or more independent experiments were performed including three mice per group with similar results.

### Effects of anti-LFA-1 treatment on anti-viral T_EFF_ differentiation

**Figure 2.**
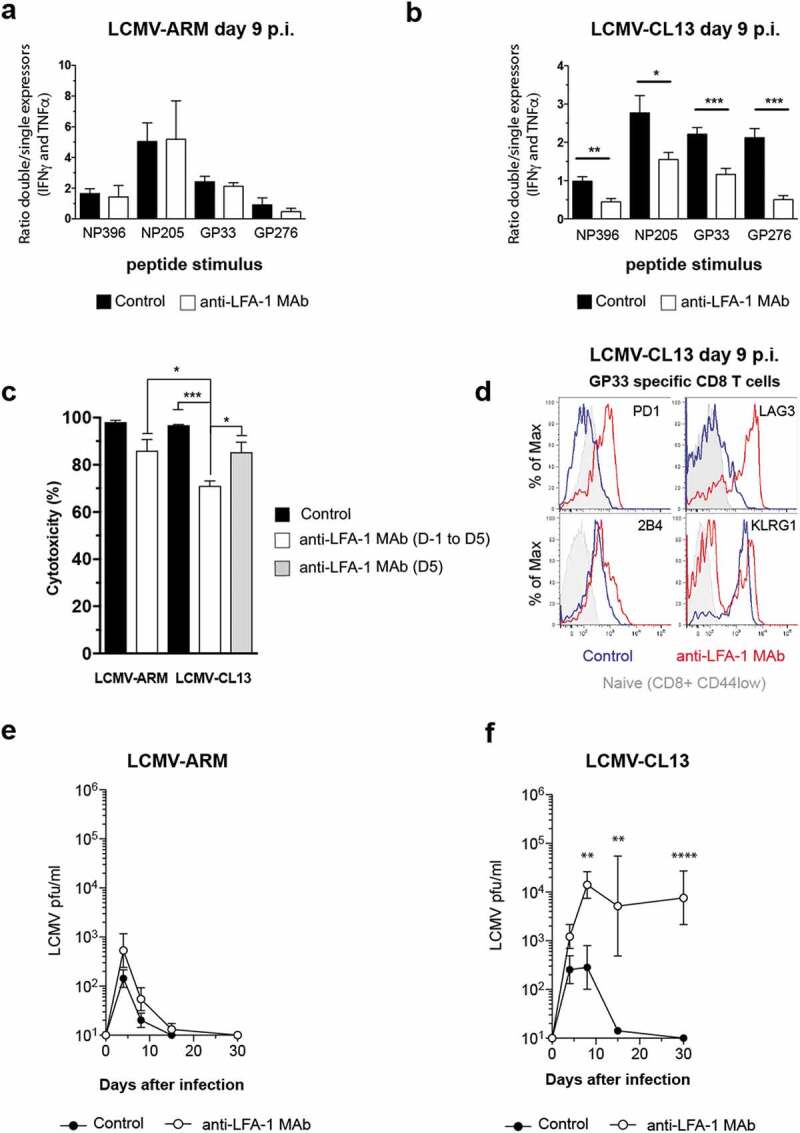
Strain-specific effects of anti-LFA-1 Mab treatment on anti-viral T_EFF_ differentiation

The efficiency of anti-viral CTL immunity is thought to be dictated by two key parameters: the T_EFF_ burst size and the ability of T_EFF_ to produce cytokines and kill infected cells. Hence, we asked if LFA-1 also impacts CD8 T_EFF_ differentiation at the single-cell level by quantifying the frequency of virus-specific T_EFF_ that co-produce IFNγ and TNFα. Although anti-LFA-1 Mab markedly reduced the overall frequency of cytokine-producing cells, the ratio of the remaining T_EFF_ that co-expressed IFNγ and TNFα or only one of these cytokines, an indicator of CTL activity [20,23], remained unchanged in LCVM-ARM infected mice (Figure 2(a)). In contrast, when mice were infected with the same dose of LCMV-CL13, the remaining CD8 T_EFF_ were preferentially impaired in their ability to co-express IFNγ and TNFα regardless of the viral peptide they recognized (Figure 2(b)). Even though LFA-1 is known to participate in immune synapse formation and the response to antigen stimulation, this strain-specific effect observed on cytokine production excludes the possibility that T_EFF_ are not able to respond to the restimulation assay due to the presence blocking antibody. Similar results were obtained using an *in vivo* killing assay [24], where cytotoxicity was significantly reduced in anti-LFA-1 Mab treated mice infected with LCMV-CL13, but not with LCMV-ARM (Figure 2(c)). Moreover, acute LFA-1 Mab treatment at day 5 p.i. only mildly affects CTL activity in LCMV-CL13 infected animals, thus indicating that the reduced CTL function that we observe is not due to the inability of CD8 T_EFF_ to respond to our assay. Altogether, LFA-1 has previously been shown to regulate the formation of a killer synapse between T_EFF_ and target cells or homing of T_N_ to lymphoid organs, and all of these effects may contribute to compromise anti-viral CD8 T_EFF_ activity. However, the observed strain-specific effect of anti-LFA-1 Mab cannot be entirely attributed to these mechanisms since all of these steps were equally inhibited in both infections.

(a,b) Differentiation of CD8 T_EFF_ into cytokine producer cells evaluated by *ex vivo* restimulation with different LCMV peptides at day 9 p.i. Results are displayed as the ratio between the frequency of IFNγ^+^ TNFα^+^ double-positive cells and the sum of IFNγ^+^ and TNFα^+^ single positive cells among CD8+ cells isolated from the blood of infected mice. (c) *In vivo* cytotoxicity capacity of GP33-specific T_EFF_ was evaluated by the differential killing of GP33 peptide-loaded splenocytes in comparison to control non-loaded splenocytes upon i.v. injection into LCMV-infected mice at day 6 p.i. (d) Surface expression of PD-1, LAG-3, 2B4, and KLRG1 in LCMV GP33-specific CD8 T_EFF_ isolated day 9 p.i. from the blood of LCMV-CL13 infected mice. (e-f) The capacity of LCMV-infected mice to control viral replication upon treatment with anti-LFA-1 Mab. Results show blood viremia. Detection limit 100pfu. See also Figure S2. * p < 0.05, ** p < 0.001, *** p < 0.0001. Error bars represent mean±SEM. Three or more independent experiments were performed including three mice per group with similar results.

CD8 T_EFF_ dysfunction may be accompanied by the expression of inhibitory molecules, which dampen anti-viral activity [25]. Accordingly, on day 9 post-LCMV-CL13 infection, virus-specific CD8 T_EFF_ from anti-LFA-1 Mab treated mice had markedly upregulated PD1, LAG3, and 2B4, while virus-specific CD8 T_EFF_ from infected control mice expressed moderate levels of 2B4, but not PD-1 or LAG3 (Figure 2(d)). In contrast, KLRG1 failed to be upregulated on CD8 T_EFF_ during LFA-1 Mab treatment to the levels of CD8 T_EFF_ from control mice, indicating a less advanced T_EFF_ differentiation [26]. Despite the reduced effector burst, anti-LFA-1 Mab treatment did not compromise the ability of mice to control LCMV-ARM (Figure 2(e)). In contrast, the control of LCMV-CL13 infection was markedly impaired after anti-LFA-1 Mab treatment and resulted in more severe disease, as evidenced by increased loss of body weight (Figure 2(f) **& S2**).

### Role of LFA-1 in CD8 T_EFF_ cell differentiation and function

**Figure 3.**
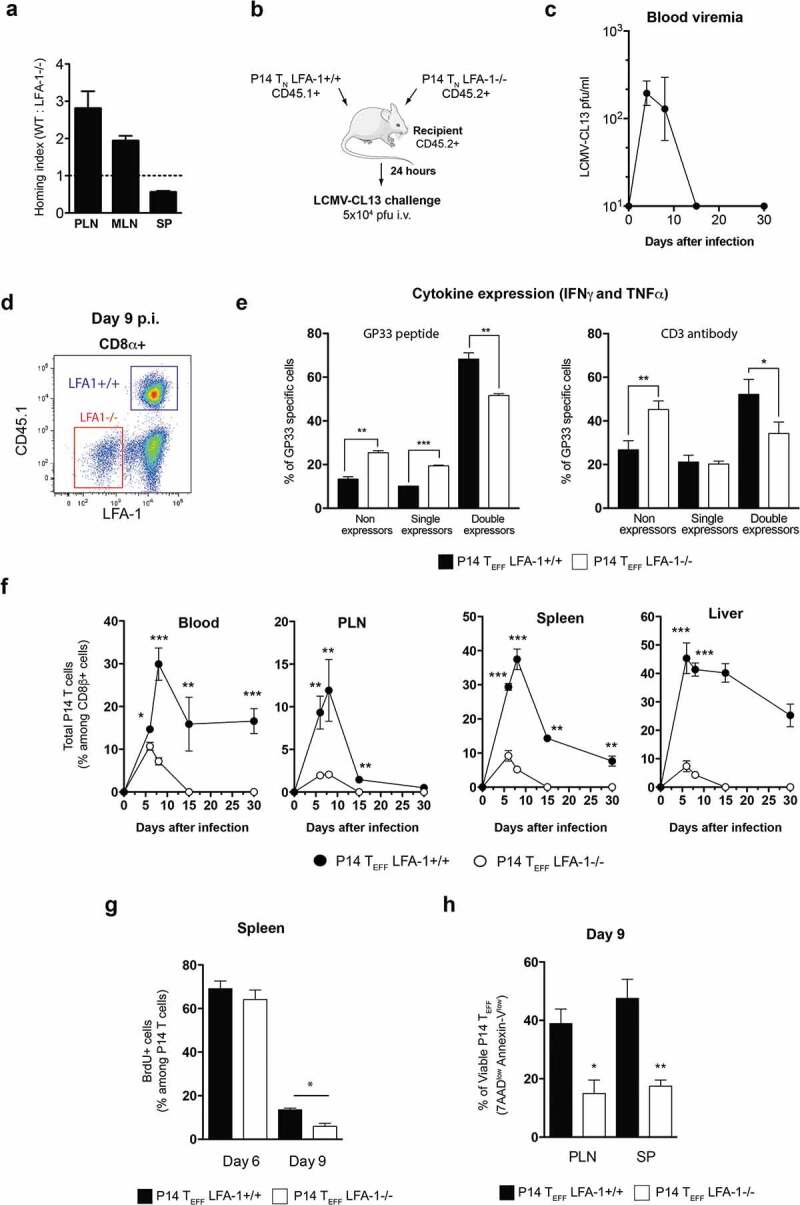
Impact of LFA-1 on CD8 T_EFF_ differentiation and function after LCMV-CL13 infection

So far our results indicate a fundamental role for LFA-1 in the CD8 T_EFF_ effector burst size in both LCMV-ARM and CL13 infection. However, CTL function is mainly affected by LFA-1 Mab treatment in the context of LCMV-CL13 infection, which cannot be cleared by infected animals. Hence, CTL dysfunction may arise from a lack of LFA-1 usage and/or increase viremia. To further explore the role of LFA-1 in CD8 T_EFF_ function during an LCMV-CL13 infection, we compared the anti-viral response kinetics of adoptively transferred CD8^+^ T_N_ from LFA1^+/+^ and LFA-1^−/-^ donors that had been crossed to P14 mice, which express atransgenic TCR that recognizes an immunodominant epitope of LCMV, gp33-41, in H-2 D^b^ [27]. When LCMV-CL13 is inoculated intravenously, the infection rapidly spreads throughout the body. However, LFA-1 is required for optimal T_N_ homing to LNs [28,29]. Accordingly, when equal numbers of differentially labeled LFA-1^+/+^ and LFA-1^−/-^P14 T_N_(2–6x10^6^ cells) were injected intravenously (i.v.) into C57BL/6 mice, the number of LFA-1^−/-^T_N_ that were recovered from recipient LNs 24 h later was ~3-fold lower than that of LFA-1^+/+^T_N_ (Figure 3(a)). This homing defect of LFA-1^−/-^P14 T_N_ was more pronounced in peripheral LNs (PLNs) than in mesenteric LNs (MLNs), while spleen homing was not compromised, consistent with the differential requirement for LFA-1 to access these lymphoid organs [1]. However, it must be cautioned that superphysiologic T_N_ precursor frequencies can alter the proliferative expansion and differentiation of T_EFF_ during an infection, presumably due to accelerated pathogen clearance [26,30]. To address this concern, we adoptively transferred smaller numbers (1x10^4^) of LFA-1^+/+^(CD45.1^+^) and LFA-1^−/-^P14 T_N_(CD45.2^+^) into CD45.2^+^C57BL/6 recipients and tracked their response (by gating on CD45.1^+^ and LFA-1^−/-^ Tcells, respectively) to LCMV-CL13 challenge (Figure 3(b)). This number of transferred T_N_ is thought to respond to LCMV equivalently to endogenous polyclonal Tcells [26]. We used the i.v. route to challenge recipients with LCMV-CL13(5x10^4^ pfu) because T_N_ responses to circulating LCMV occur mainly in the spleen [31], where LFA-1 is not required for T_N_ homing. Thus, equal numbers of both T_N_ subsets could be transferred without compromising access of either subset to viral Ag. This strategy ensures that P14 T_EFF_ are exposed to the same environment and to aviremia similar to non-chimeric C57BL/6 mice, thus allowing us to identify the role of LFA-1 in CTL function (Figure 3(c)).

Differentially labeled WT and LFA-1^−/-^ P14 T_N_ were adoptively transferred to C57BL/6 mice and homed cells in lymphoid tissues were compared at steady-state (a) and WT and LFA-1^−/-^ P14 T_N_ expansion and differentiation and viremia was followed after i.v. infection with 5 × 10^4^ pfu LCMV-CL13 (b-f). (a) Homing indices (i.e. the ratio of WT:LFA-1^−/-^ T_N_ in a recipient organ relative to input ratio 24 hours after T_N_ transfer. (b) A small number (10,000 cells) of naïve LFA-1^+/+^ CD45.1^+^ and LFA-1^−/-^ CD45.2^+^ P14 T_N_ cells were enriched by magnetic negative selection (>95% purity) and transferred into the same WT CD45.2^+^ recipient, thus generating P14 chimeric mice. (c) 24 hours later, recipient mice were i.v. challenged with LCMV-CL13 (5x10^4^ pfu) that were able to clear infection with kinetics similar to non-chimeric mice. See also Figure 2c. (d) The surface expression of CD45.1 and lack of LFA1 expression was used to identify adoptively transferred P14 T_EFF_ cells among CD8+ cells in recipient mice. (e) On day 9 p.i. the expression of cytokines (IFNγ and TNFα) by individuals LFA1^+/+^ and LFA1^−/-^ P14 T_EFF_ cells was determined after *ex vivo* restimulation with gp33-41 or soluble CD3 antibody of splenocytes from LCMV-CL13 infected mice (d) At different times after infection the T_EFF_ burst size generated by adoptively transferred T_N_ P14 was monitored in lymphoid and non-lymphoid organs of P14 chimeric mice by flow cytometry. (e) During the expansion phase (day 6 p.i.) and at the beginning of the contraction phase (day 9 p.i.) the rate of proliferation of LFA1^+/+^ and LFA1^−/-^ T_EFF_ was quantified by the incorporation of BrdU into dividing cells. BrdU was injected i.p. in LCMV-CL13 infected mice and 12 h later its incorporation into LFA1^+/+^ and LFA1^−/-^ T_EFF_ was quantified by flow cytometry. (f) At day 9 p.i. splenocytes and lymph node cells of LCMV-CL13 infected mice were harvested and the binding to Annexin-V and incorporation of 7AAD into LFA1^+/+^ and LFA1^−/-^ T_EFF_ was quantified by flow cytometry. See also Fig. S3&4. * p < 0.05, ** p < 0.001, *** p < 0.0001. Error bars represent mean±SEM.Three or more independent experiments were performed including three mice per group with similar results.

**Figure 4.**
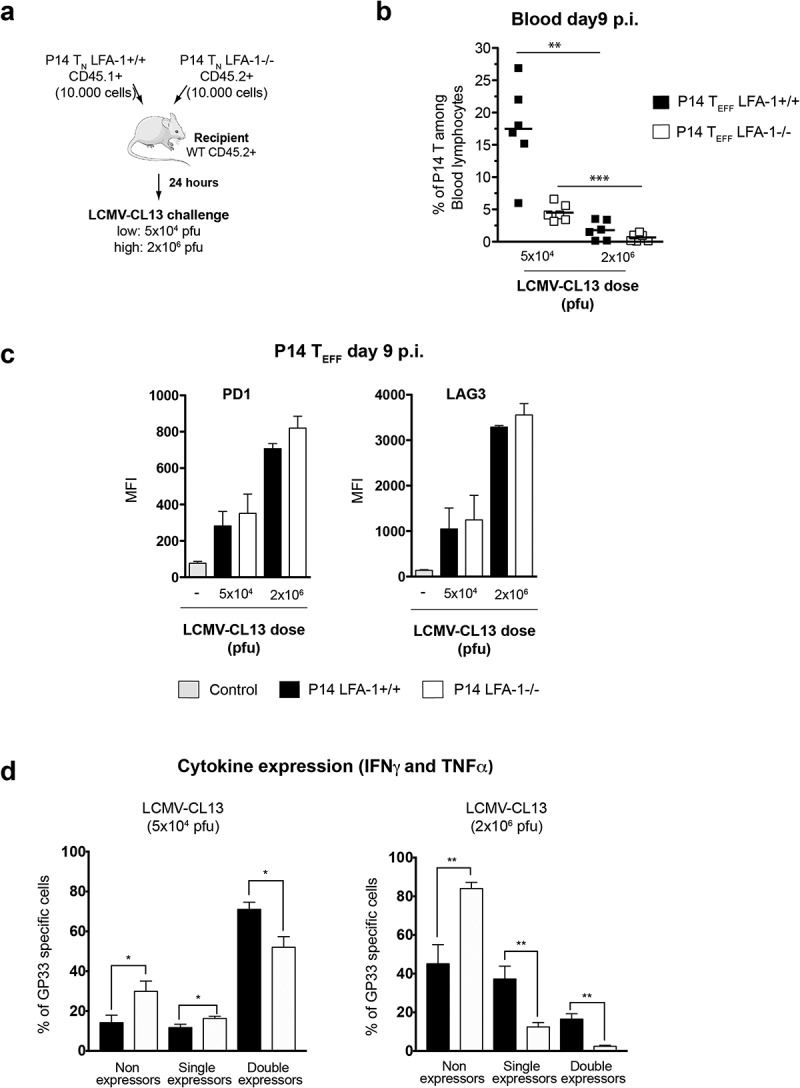
**Role of LFA-1 usage and high viremia in T_EFF_ dysfunction after LCMV-CL13 infection** (a) 10,000 cells of purified LFA-1^+/+^ CD45.1^+^ and LFA-1^−/-^ CD45.2^+^ P14 T_N_ cells were transferred into the same C57BL/6 CD45.2^+^ recipients, thus generating P14 chimeric mice. 24 hours later, recipient mice were i.v. challenged with LCMV-CL13 (5x10^4^ or 2 × 10^6^ pfu). The surface expression of CD45.1 and lack of LFA1 expression was used to identify these cells among CD8+ cells in recipient mice. At day 9 p.i. the frequency (b) and (c) the expression of PD-1 and LAG-3 was quantified in P14 T_EFF_ from the blood of infected P14 chimeric mice. Results shown as mean fluorescence intensity (MFI). (d) In parallel, P14 T_EFF_ from the blood of infected mice were restimulated *ex vivo* with LCMV GP33 peptide to quantify their production of IFNγ and TNFα. Two independent experiments were performed including three mice per group with similar results

After LCMV-CL13 (5x10^4^ pfu) intravenous infection, P14 T_EFF_ were identified by their expression of CD45.1 or lack of LFA-1 among CD8 T cells (Figure 3(d)). Our results show that the cytokine profile of late (day 9) T_EFF_ derived from LFA-1^−/-^ P14 T_N_ that had been transferred at near-physiological numbers was compromised; compared to LFA-1^+/+^ P14 T_EFF_, the fraction of LFA-1^−/-^ P14 T_EFF_ that did not express cytokines after peptide stimulation was moderately increased and the fraction that co-expressed two cytokines was reduced (Figure 3(e)). Moreover, this effect could also be observed after stimulation with soluble CD3 antibody a setting where LFA-1’s role in cell–cell interactions is not required. Hence, poor co-expression of cytokines cannot be explained by a lack of response of LFA-1^-/-^ P14 T_EFF_ to our assay. This effect was paralleled by phenotypic differences between LFA-1^+/+^ and LFA-1^−/-^ P14 T_EFF_ in lymphoid organs consistent with compromised terminal differentiation of the latter, as evidenced by attenuated downregulation of CD27 and CD62L and ~30% reduced frequency of LFA-1^−/-^ P14 T_EFF_ cells that had upregulated KLRG1 (**Fig. S3**). While these phenotypic and functional effects of LFA-1 deficiency were relatively subtle, the impact on P14 T_EFF_ numbers was much more dramatic; in all organs studied the number of LFA-1^−/-^ P14 T_EFF_ was profoundly reduced as compared to LFA-1^+/+^ P14 T_EFF_ cells (Figure 3(f)). This suggests that the decrease in effector burst size of LFA-1^−/-^ P14 T_EFF_ cells was not a consequence of defective intravascular lymphocyte trafficking to secondary lymphoid organs. Furthermore, the differences in burst size between LFA-1^+/+^ and LFA-1^−/-^ T_EFF_ P14 cells could also be observed when P14 T_N_ are adoptively into separate recipients infected with LCMV-CL13 (**Figure S4**). Hence, excluding the possibility that the competition between LFA-1^+/+^ and LFA-1^−/-^ T_N_ P14 cells for APCs may explain the differences in P14 T_EFF_ effector burst size.

The overall T_EFF_ burst size is determined, in part, by the rate of T cell proliferation and, in part, by the rate of cell death. Following LCMV-CL13 infection, the rate of cell division was similar between LFA-1^+/+^ and LFA-1^−/-^ P14 T_EFF_ at the peak of T cell expansion (day 6), but decreased more rapidly in LFA-1^−/-^ P14 T_EFF_ during the early steps of the contraction phase on day 9 (Figure 3(g)). As expected, contraction of the CTL response on day 9 p.i. caused a sizable fraction of both LFA-1^+/+^ and LFA-1^−/-^ P14 T_EFF_ to display apoptosis markers, including Annexin-V binding and incorporation of 7AAD. However, the percentage of viable cells (Annexin-V^low^ 7AAD^low^) was significantly lower among LFA-1^−/-^ P14 T_EFF_ (17 ± 2%) as compared to LFA-1^+/+^ P14 T_Eff_ (47 ± 6%), suggesting that the absence of LFA-1 predisposes T_EFF_ to undergo accelerated death (Figure 3(h)).

### LFA-1 protects against CD8 T_EFF_ dysfunction but not upregulation of inhibitory receptors

To further identify the contribution of the loss of LFA-1 function and high viremia toward CD8 T_EFF_ dysfunction we employed the same strategy as before to generate P14 chimeric mice; however, in this setting mice were challenged with either 5 × 10^4^ or 2 × 10^6^ pfu of LCMV-CL13 (Figure 4(a)). Whereas a 5 × 10^4^ pfu challenge will result in an acute infection, a 2 × 10^6^ pfu challenge will lead to a chronic infection. We found that a challenge with 2 × 10^6^ pfu of LCMV-CL13 was associated with reduced P14 T_EFF_ cell numbers and higher expression levels of PD-1 and LAG-3 in comparison to a 5 × 10^4^ pfu challenge, but these effects were independent of LFA-1 usage (Figure 4(b, c)). Higher viremia also impaired the capacity of P14 T_EFF_ to co-produce IFNγ and TNFα, but these effects were exacerbated in LFA1^−/-^ P14 T_EFF_. Indeed, the vast majority of LFA1^−/-^ P14 T_EFF_ (81 ± 2%) were unable to produce either IFNγ or TNFα (Figure 4(d)). Consistent with LFA-1’s role in CD8 T_EFF_ dysfunction impaired cytokine production can be also observed in LFA1^−/-^ P14 T_EFF_ upon challenge with 5 × 10^4^ pfu LCMV-CL13.

### Transcriptome analysis of CD8 T_EFF_ dysfunction in the absence of LFA-1 function

In order to define the underlying pathways resulting in CD8 T_EFF_ dysfunction in the absence of LFA-1 function (anti-LFA-1 Mab treatment and genetic deficiency) in mice challenged with LCMV-CL13, we performed a global transcriptome analysis of LCMV-specific CD8 T_EFF_. This analysis was performed in two settings in order to dissociate the impact of LFA-1 usage from exposure to high viremia in our transcriptome analysis: first, we sorted from anti-LFA-1 Mab treated and control mice endogenous LCMV GP33 specific (Dex-GP33) T_EFF_ at day 9 p.i with LCMV-CL13 (5x10^4^ pfu); second, we adoptively transferred 10^4^ cells of LFA-1^+/+^ and LFA-1^−/-^ P14 T_N_ into C57BL/6 mice (P14 chimeric mice) and challenged them with LCMV-CL13 (5x10^4^ pfu). Next, we sorted at day 9 p.i. from LCMV-CL13 infected mice LFA-1^+/+^ and LFA1^−/-^ P14 T_EFF_ (**Fig. S5**). Importantly, only in the former setting viremia is controlled by the recipient mice.

**Figure 5.**
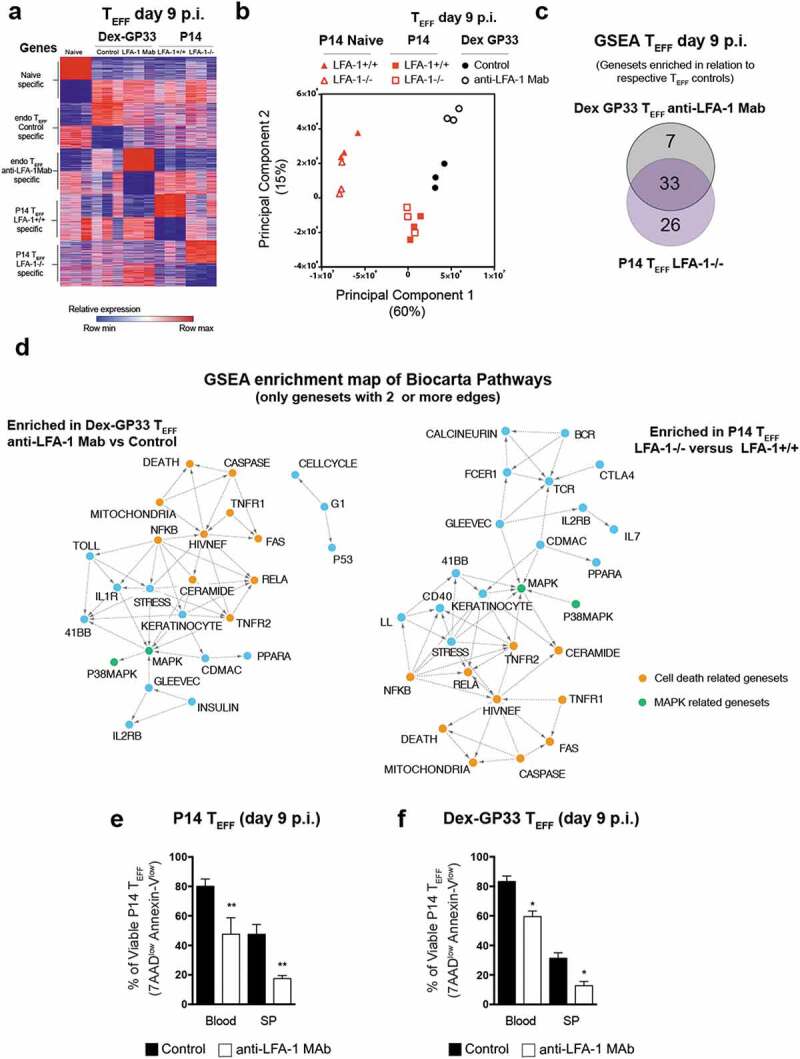
**Global transcriptomics of T_EFF_ at the peak of anti-viral effector response** (a) Genes differentially expressed in GP33-specific T_N_ and T_EFF_ from anti-LFA-1 Mab antibody and control treated mice. Each column represents an individual sample and each row a gene, and cells were colored to indicate relative expression. Top 200 genes upregulated or downregulated in each specific population are shown. (b) Principal Component Analysis of transcriptomic profiles of T_N_ and T_EFF_. (c) Gene set enrichment analysis was performed between endogenous Dex-GP33 T_EFF_ from anti-LFA-1 Mab treated versus control mice and LFA-1^+/+^ versus LFA-1^−/-^ P14 T_EFF_. Overlap of enriched pathways are shown as a Venn diagram. (e,f) At day 9 p.i. splenocytes and blood cells of LCMV-CL13 infected mice were harvested and the binding to Annexin-V and incorporation of 7AAD into T_EFF_ was quantified by flow cytometry. Three independent experiments including three mice per group were performed with similar results

As expected, at day 9 p.i. both P14 and Dex-GP33 T_EFF_ acquired a transcriptome profile distinguishable from T_N_ (Figure 5(a)). A Principal Component Analysis (PCA) of our data shows 2 principle components (PC1&2) account for the majority of the variability in our data (75%) and reveals 4 clusters (T_N_, P14T_EFF_, Dex GP33 control and Dex-GP33 anti-LFA-1). Moreover, it shows that anti-LFA-1 Mab treatment and high viremia significantly perturbed Dex-GP33 T_EFF_, whereas the impact of the loss of LFA-1 during an acute infection was less pronounced (Figure 5(b)). To better define the impact of LFA-1 on CD8 T_EFF_ function we performed a Gene Set Enrichment Analysis (GSEA) using BIOCARTA curated gene sets from the Molecular Signatures Database (MSigDB v7.1). Our goal was to identify the pathways that were perturbed in Dex-GP33 T_EFF_ from anti-LFA-1 Mab treated mice and LFA-1^−/-^ P14 T_EFF_ in comparison to Dex-GP33 T_EFF_ from control mice and LFA-1^+/+^ P14 T_EFF,_ respectively. Our results show that impaired LFA-1 function significantly altered 40 pathways in Dex-GP33 T_EFF_ from anti-LFA-1 Mab treated mice and 59 pathways in LFA-1^−/-^ P14 T_EFF_ (False Discovery Rate (FDR)<0.2 and p < 0,05) (**Table S1&2**). Among these pathways, 33 were shared by the 2 subsets, which should be impacted by the loss of LFA-1 function but not high viremia (Figure 5(c)**, S6**). An Enrichment map analysis of these results show that a major theme linked to loss of LFA-1 function is cell death represented by the Caspase, Death, TNFR1&2, Fas, Ceramide, RelA, NfkB pathways (Figure 5(d)). Consistent with the fact that Dex-GP33 T_EFF_ from anti-LFA-1 Mab treated mice and LFA-1^−/-^ P14 T_EFF_, show a lower number of viable cells (Annexin-V^low^, 7AAD^low^) in comparison to their respective T_EFF_ controls (Figure 5(e,f)). Accordingly, we found that *casp3* and *8* were significantly altered by anti-LFA-1 Mab treatment in Dex-GP33 T_EFF_ and to a lower extent in LFA-1^−/-^ P14 T_EFF_ (**Fig. S7a,b**). These results suggest that a lower CD8 T_EFF_ burst size in the absence of LFA-1 function is due to a perturbation of TNF/FAS pathways, in line with the fact that TNF and Fas pathways regulate cell death in CD8 T cells [32,33]. Moreover, HIV/NEF, MAPK and RelA gene-sets share features with cell death-related gene-sets suggesting a possible role for these pathways in the lower CD8 T_EFF_ burst size in the absence of LFA-1 function (Figure 5(d)). Our transcriptomic analysis also showed significant differences between Dex-GP33 T_EFF_ from anti-LFA-1 treated mice and LFA-1^−/-^ P14 T_EFF_ (**Fig. S6**). Indeed, Dex-GP33 T_EFF_ isolated from anti-LFA-1 Mab treated mice showed the upregulation of several gene-sets, including IL-1R and IL-10 that are implicated in LCMV induced wasting disease [34] and control of viremia [35]. On the other hand, several pathways specific to LFA-1^−/-^ P14 T_EFF_ are related to TCR signaling (RAS, AKT, calcineurin, MTOR, ARAP), consistent with LFA-1’s role in the immune synapse.

**Figure 6.**
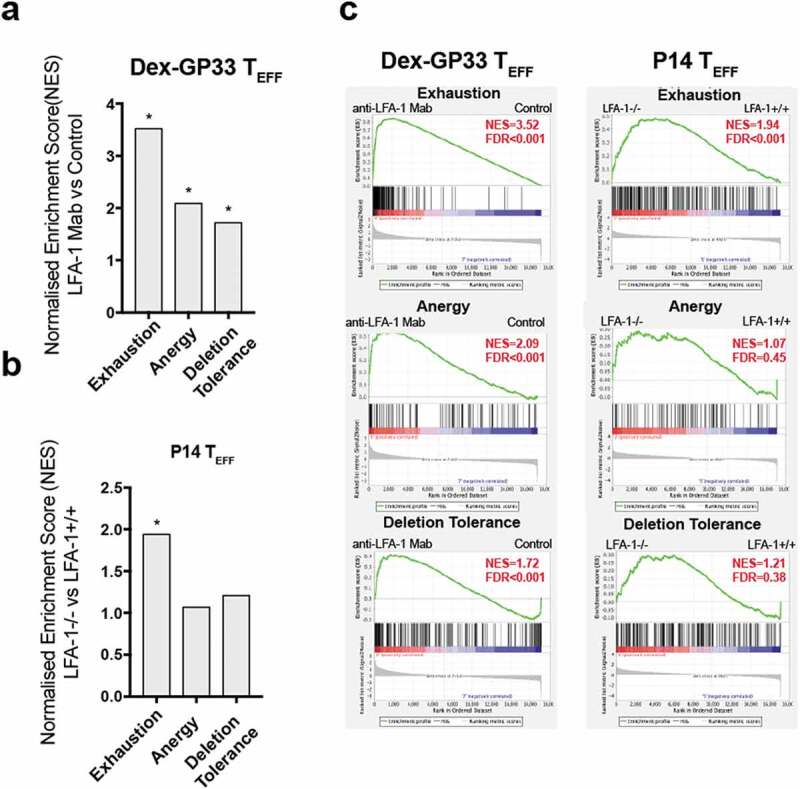
**Transcriptional signatures of exhaustion, anergy and deletion tolerance in T_EFF_ in the absence of LFA-1 function**. Geneset enrichment analysis (GSEA) on T cells exhaustion [41], anergy [40] and deletion tolerance [39] signatures from MSigDB v7.1 (Broad institute) was performed on day 9 Dex-GP33 (a,c) or P14 T_EFF_ (b,c) from LCMV-Cl13 infected mice. (*) represents signatures with a false discovery rate (FDR) of <0.05

In addition to a perturbation in cell death pathways, our results show that in the absence of LFA-1 function CD8 T_EFF_ are unable to co-express cytokines upon restimulation, have poor CTL activity, and upregulate inhibitory receptors, some of the hallmarks of T cell exhaustion [25]. However, exhausted CD8 T cells are primed properly [25] whereas in the absence of LFA-1, which is known to enhance TCR signaling [36,37], CD8 T_N_ cells receive a suboptimal stimulation that may produce a state of tolerance leading to deletion or anergy [38]. Hence, to understand the type of dysfunction that is induced in CD8 T_EFF_ in the absence of LFA-1 we have compared the transcription state of endogenous GP-33 specific T_EFF_ from mice treated with anti-LFA-1 Mab and LFA-1^-/-^ P14 T_EFF_ to molecular signatures of deletion tolerance, anergy, and exhaustion on CD8 T cells [39–42]. Even though LFA-1 function has been extensively studied, a global analysis of its role in CD8 T cells function by transcriptomics has not yet been performed. Interestingly, LFA-1 is among the genes of a transcriptional signature of exhaustion and deletion tolerance, implying that it may be involved in both processes [39,41]. Our results show that endogenous GP-33 T_EFF_ from mice treated with anti-LFA-1 Mab acquired a transcriptomic signature of exhaustion (NES = 3.52) and to a lesser extent a signature of anergy (NES = 2.09) and deletion tolerance (NES = 1.72) (Figure 6(a,c) **& Fig.S8**). In contrast, LFA-1^-/-^ P14 T_EFF_ cells acquired only an exhaustion signature (NES = 1.94) and to a lesser degree than GP-33 T_EFF_ from mice treated with anti-LFA-1 Mab (Figure 6(b,c) **& Fig. S9**). These results are in line with the perturbations of *tbx2 and eomes* expression in Dex-GP33 T_EFF_ from mice treated with anti-LFA-1 Mab since *tbx21* represses, whereas *eomes* promotes, the expression of exhaustion markers [43,44] (**Fig. S10a&b**). Furthermore, GP-33 T_EFF_ from mice treated with anti-LFA-1 Mab show increased *egr2* expression, a transcription factor that is upregulated in anergic and exhausted CD8 T cells [40,42], which may explain the fact that these cells show a signature reminiscent of both dysfunctional states. However, LFA-1^−/-^ P14 T_EFF_ were not yet fully committed to an exhausted phenotype, since they show a mild upregulation of PD-1, LAG3, and GP49 inhibitory receptors in comparison to Dex-GP33 T_EFF_ from mice treated with anti-LFA-1 Mab (**Fig. S11a&b**). Moreover, LFA-1^−/-^ P14 T_EFF_ did not show deficiencies in the expression of genes involved in cytotoxic activity, besides GzmA (**Fig. S12a, b)**. Hence, poor CTL activity in LFA-1^−/-^ P14 T_EFF_ cannot be explained entirely by a lack of expression of CTL genes, similar to exhausted CD8 T cells [41]. Altogether, our results show that lack of LFA-1 function during LCMV-CL13 infection seems to drive, in part, a transcriptional signature of exhaustion in T_EFF_, which in the presence of persistent viremia can additionally trigger an anergy and deletion tolerance signatures. However, the lack of LFA-1 function is not sufficient to trigger all aspects of any of these dysfunctional states; thus, its absence may only trigger a CTL dysfunctional state that predisposes to T_EFF_ dysfunction if viremia persists.

## Discussion

In light of the critical contribution of LFA-1 to leukocyte-mediated inflammation in animal models, considerable efforts were made to develop inhibitors of LFA-1 function as a therapy for inflammatory diseases in humans. These activities ultimately led to the introduction of efalizumab, a humanized monoclonal antibody (MAb) to the β subunit of LFA-1, that is efficacious in moderate to severe psoriasis [45]. However, some recipients of efalizumab developed progressive multifocal leukoencephalopathy (PML), an often fatal opportunistic infection of the CNS caused by JC virus [10,46]. These clinical observations suggest that LFA-1 plays an important role in host resistance to certain viral infections by mechanisms that are still incompletely understood. Since circulating antibodies against JC virus do not appear to protect against the development of PML [47], it is likely that the LFA-1-dependent protection involves cell-mediated anti-viral effector responses. Thus, the present study was undertaken to specifically explore the role of LFA-1 during a viral infection that requires a cellular immune response. Specifically, we focused on murine host responses to an acute infection with lymphocytic choriomeningitis virus (LCMV), which depends on the generation of CD8^+^ effector T cells (T_EFF_) for viral clearance.

The results presented here challenge the established view that LFA-1 function is not required for the control of an LCMV infection in a mouse model. This paradigm was established in studies where LFA-1^−/-^ or ICAM1^−/-^ mice were challenged with LCMV-ARM but failed to reveal any major role for CD8 T cell immunity, which is critical to control infection [48]. However, these results created a conundrum due to the compelling clinical data showing predisposition to infection upon loss of LFA-1 function. Indeed, leukocyte adhesion deficiency-1 syndrome (deficiency in β2 integrins) [49] and treatment of patients with a humanized anti-LFA-1 Ab predispose to infection [46].

We believe that our work sheds a new light into this conundrum. Unlike other studies that rely on a systemic LFA-1-deficiency of the host, our approach was based on blocking LFA-1 function at defined steps of infection or specific deletion of LFA-1 on CD8 T cells in order to identify the key mechanisms involved in susceptibility to infection. Using this approach, we found that inhibition of LFA-1 during the entire anti-viral effector phase (until day 7 post-infection) impaired the magnitude of the CD8 T_EFF_ response independently of the virulence of the LCMV strain used for challenge (ARM or CL13). In contrast, CD4 T cell immunity was unaffected by this treatment, suggesting a cell-specific requirement for LFA-1 in CD8 T_EFF_ expansion. Furthermore, the impact of LFA-1 inhibition in CD8 T_EFF_ burst size cannot be explained entirely by an impairment in T_EFF_ homing or T cell–APC interactions since this process is inhibited in both LCMV-CL13 and LCMV-ARM infections. To better understand the role of LFA-1 on CD8 T_EFF_ cells we performed a global transcriptomic analysis, which even though LFA-1 function has been extensively studied, this type of analysis had not yet been performed. Transcriptome analysis revealed that CD8 T_EFF_ generated in the absence of LFA-1 had significant alteration in several pathways involved in cell death. Indeed, several pathways (Caspase, Death, TNFR1&2, Fas, Ceramide, RelA, NfkB) that regulate T_EFF_ survival [32,33], were considerably affected in T_EFF_ by this treatment. This gene expression profile was in line with the upregulation of markers of apoptosis (Annexin-V binding and incorporation of 7AAD) by T_EFF_ in the absence of LFA-1.

Surprisingly, a similar reduction in T_EFF_ burst size by LFA-1 inhibition had an LCMV strain-specific impact on the outcome of infection. Mice that were treated with anti-LFA-1 Mab and challenged with LCMV-ARM cleared the virus with similar kinetics to untreated mice, whereas LCMV-CL13 challenged mice developed a persistent viremia. Because LCMV-CL13 strain has a higher efficiency to infect and replicate within cells than the ARM strain, inhibition of LFA-1 increases the susceptibility to infection with a high replicating/spreading virus. This result is consistent with the idea that an excess of effector cells versus infected cells must be attained in order to prevent a chronic infection [50] and that the optimal number of effector cells is dictated by the replication/spreading capacity of the virus. Hence, the loss of LFA-1 that leads to a reduction of T_EFF_ numbers could tilt the balance in favor of LCMV-CL13 replication/spreading leading to high viremia. Such phenomenon would not occur in mice challenged with the LCMV-ARM, since it has a lower replication/spreading capacity, and therefore, a low number of effector CD8 T cells would be sufficient to eliminate the viral load. Our results are also in line with the fact that JC virus isolated from brain and cerebrospinal fluid of PML patients treated with anti-LFA-1 Mab bear mutations that confer them greater replication capacity [51], which we found to be a key factor in the predisposition to LCMV infection after LFA-1 inhibition.

The absence of LFA-1 function in CD8 T cells also triggered a dysfunctional state in the remaining CD8 T_EFF_ that lead to loss of cytokine expression, poor CTL activity, and upregulation of inhibitory receptors. CD8 T cell dysfunction can arise either from a suboptimal stimulation leading to tolerance (anergy or deletion) or from chronic exposure to Ag and inflammatory environment leading to exhaustion [25,38]. These dysfunctional states are driven by distinct transcriptional programs and develop early (tolerance) or late (exhaustion) during the immune response. Our transcriptomic analysis permitted to shed light into the type of dysfunctional state that is triggered in the absence of LFA-1 function. Indeed, P14 T_N_ cell priming in the absence of LFA-1 during LCMV-CL13 acute infection triggers a partial transcription program of T cell exhaustion. However, this exhaustion signature was incomplete and did not lead to the upregulation of inhibitory receptors during LCMV-CL13 acute infection. In contrast, CD8 T_EFF_ isolated from LCMV-Cl13 infected mice that underwent anti-LFA-1 Mab and could not control viremia simultaneously upregulated different transcription signatures of exhaustion, anergy, and deletion tolerance. These results suggest that T cell activation in the absence of LFA-1 triggers a partial exhaustion signature that is reinforced by the exposure to higher viremia, which can also lead to the acquisition of tolerance signatures. Altogether, we envision that LFA-1 plays a critical role in controlling CD8 T_EFF_ dysfunction through its role in the formation of a mature synapse and modulation of TCR signaling: first, during T_N_ priming by boosting weak and/or intermittent TCR signals and avoiding the emergence of T cell dysfunction; second, during exposure to high viremia by attenuating strong and/or repeated TCRs signaling and preventing exacerbation of T_EFF_ dysfunction [7,52]. Even though LFA-1’s role in increasing TCR signaling has been previously studied TCR [36,37], its role in the context of high Ag was not yet explored. Supporting this hypothesis, we found that LFA-1^−/-^ P14 T_EFF_ were extremely impaired in their differentiation into cytokine producers when exposed to high levels of viral antigens but upregulated inhibitory receptors similarly to LFA-1+/+ P14 T_EFF_. Hence LFA-1 plays a major role in regulating T cell dysfunction in line with the fact that this gene is part of transcriptomic signatures previous described in the context of deletion tolerance [39] and T cell exhaustion [41].

In conclusion, our study reveals an unexpected role for LFA-1 in regulating T_EFF_ dysfunction and cell death during LCMV infection. However, the protection conferred by LFA-1 against infection is dependent on the virulence of the LCMV strain, thus implying that the loss of LFA-1 function specifically increases the susceptibility to infections by highly replicating/spreading viruses. Overall, we provide new and important insights into a mechanism governing CD8 T cell function during infection that when defective can ultimately lead to an increased susceptibility to a viral infection.

## Methods

### Mice

Donor P14 and OTI transgenic mice (LFA-1^+/+^ or LFA-1^−/-^) were bred in house or purchased from Taconic Farms, respectively, while donor P14 CD45.1^+^ were provided by Liisa Selin (Umass Medical School, Worcester, MA). Recipient mice were age matched. Adoptive transfer by intravenous injection of 10^4^ P14 LFA-1^+/+^ and 10^4^ P14 LFA-1^−/-^ T_N_ cells into C57Bl/6 was used to generate P14 chimeric mice. Mice were housed under specific-pathogen-free conditions in accordance with the National Institutes of Health (NIH) guidelines.

### Ethics statement

The Institutional Animal Committees of Harvard Medical School and IDI approved all experimental animal procedures.

### Cell Purification, adoptive transfer, antibody treatment and LCMV infection

T cell purification was done by negative selection using Miltenyi MACs bead separation according to the manufacturer’s instructions and adoptive transfer performed by intravenous injection. Infection of mice was performed by low dose intravenous challenge of LCMV-ARM or CL13 using 5 × 10^4^ pfu of each strain while a high dose challenge with LCMV-CL13 was done by intravenous injection with 2 × 10^6^ pfu. LFA-1 Mab antibody (M17/4 clone, BioXcell) or PBS for treatment of infected mice was done by intraperitoneal injection of 200 μl (200 μg for M17/4) of this reagent from day −1 to 7 of LCMV infection, at the two-day interval. Virus levels were assayed by plaque assays as previously described [20]

### Lymphocyte isolation and flow cytometry

Lymphocytes were isolated from the spleen, liver, lung, lymph nodes, bone marrow, or blood followed by staining with antibodies from BD Biosciences, Biolegend, or eBioscience and the following clones: CD45.1 (A20), LFA-1 (M17/4), CD8β (53–6.7), IFNγ (XMG1.2), TNFα (TN3-19), CD4 (RM4-5), PD-1 (RMP1-30), 2B4 (2B4), LAG-3 (C9B7W), KLRG1 (2F1), IL7Rβ (A7R34), CD27 (LG.7F9), CD62L (MEL-14). Detection of endogenous GP33-specific CD8 T cells was performed using specific Dextramers (Immundex). For *ex vivo* restimulation blood lymphocytes were stimulated 5 h with LCMV peptides, CD3 antibody (1μg/ml) or no peptide in the presence of human recombinant IL2 (R&D) followed by intracellular staining for cytokines using a Fixation/Permeabilization Solution Kit (BD Bioscience). Surface and intracellular staining was performed according to the manufacturer’s instructions. Flow cytometry was performed using a FACSCanto (BD Bioscience) and analyzed using Flowjo software (Treestar).

### In vivo *cytotoxicity assay*

CFSE or CMTMR labeled splenic cells were either pulsed with 1 *μ*M GP33 peptide or left unpulsed for 1 hr at 37°C as previously described in [53]. The cells were then washed and mixed at a 1:1 ratio, and 10^7^ cells (i.e. 5 × 10^6^ CFSE+, 5 × 10^6^ CMTMR+ cells) were adoptively transferred by intravenous injection into LCMV-infected mice. Two hours post-transfer, the spleens were removed from the mice and GP33-specific cytotoxicity quantified as previously described [24].

### *Proliferation by* in vivo *BrdU incorporation*

For assessment of T_EFF_ proliferation by BrdU, mice were given 1 mg of BrdU intraperitoneally at day 5 and 8 p.i. and sacrificed 12–16 h hr later. BrdU staining was carried out with the APC BrdU Kit (BD Biosciences) according to the manufacturer’s instructions.

### Apoptosis staining

Annexin V and 7-AAD staining were performed using PE Annexin V Apoptosis Detection Kit I (BD PharMingen) according to the manufacturer’s instructions.

### Microarray analysis, normalization and data analysis

RNA was prepared from cell populations sorted with Trizol reagent as described [54]. RNA was amplified and hybridized on the Affymetrix Mouse Gene 1.0 ST array according to the manufacturer’s procedures. Raw data for all populations were preprocessed and normalized by the robust multi-array average algorithm [55] implemented in the “Expression File Creator” module in the GenePattern suite [56]. RNA processing and microarray analysis with the Affymetrix MoGene 1.0 ST array was prepared according to standard operating procedures of the ImmGen Project (http://www.immgen.org/Protocols/Total RNA Extraction with Trizol.pdf; http://www.immgen.org/Protocols/ImmGen QC Documentation_ALL-DataGeneration_0612.pdf).

Gene Set Enrichment Analysis (GSEA) was performed using software previously described [57,58] and the Molecular Signatures Database (MSigDB) was used to obtain a collection of annotated gene sets for use in this software. Unless stated otherwise all data analysis was performed using the Genpattern software (Broad Institute) [56].

## Supplementary Material

Supplemental Material

## Statistical analysis

Results are expressed as mean ±SEM unless stated otherwise. All statistical analyses were performed in Prism (GraphPad Software). Means between two groups were compared with two-tailed t-test. * p<0.05, ** p<0.001, *** p<0.0001.
