# Supplementary material for: Role of LFA-1 integrin in the control of a lymphocytic choriomeningitis virus (LCMV) infection
Source: Virulence. 2020 Nov 29;11(1):1640–55. doi: 10.1080/21505594.2020.1845506 (PMC7714442; doi:10.1080/21505594.2020.1845506)
Supplement: Supplemental Material [file KVIR_A_1845506_SM0703.docx]

**Role of LFA-1 integrin in the control**

**of a lymphocytic choriomeningitis virus (LCMV) infection**

**Supplementary Material**

**SUPPLEMENTAL FIGURES**

**
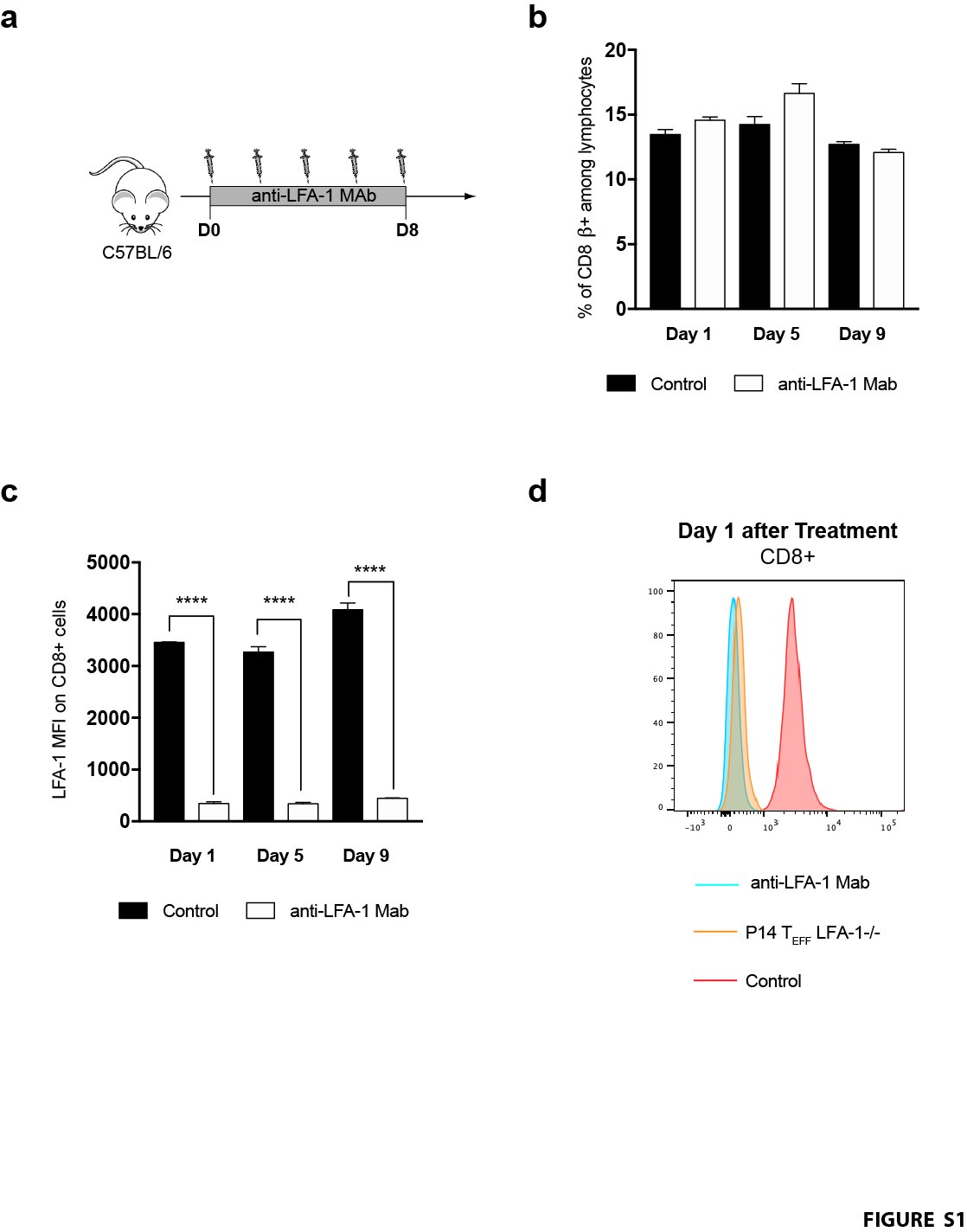
**

**Figure S1. Impact of anti-LFA1 Mab treatment in non-infected mice.**

(a) C57Bl/6 mice were treated with 200μg anti-LFA-1 Mab (BioXcell, clone M17/4) every 2 days for 8 consecutive days. During anti-LFA1 Mab treatment blood CD8+ T cells were isolated and their numbers (b) and expression levels of LFA-1 were quantified by flow cytometry (c,d). In order to detect the amount of free LFA-1 molecules in CD8 T cell isolated from anti-LFA1 treated mice we performed an antibody staining with the same clone (M17/4) that was used for treatment. As a control we used day 9 LFA1-/- P14 T_EFF_ isolated from LCMV-CL13 mice.

**
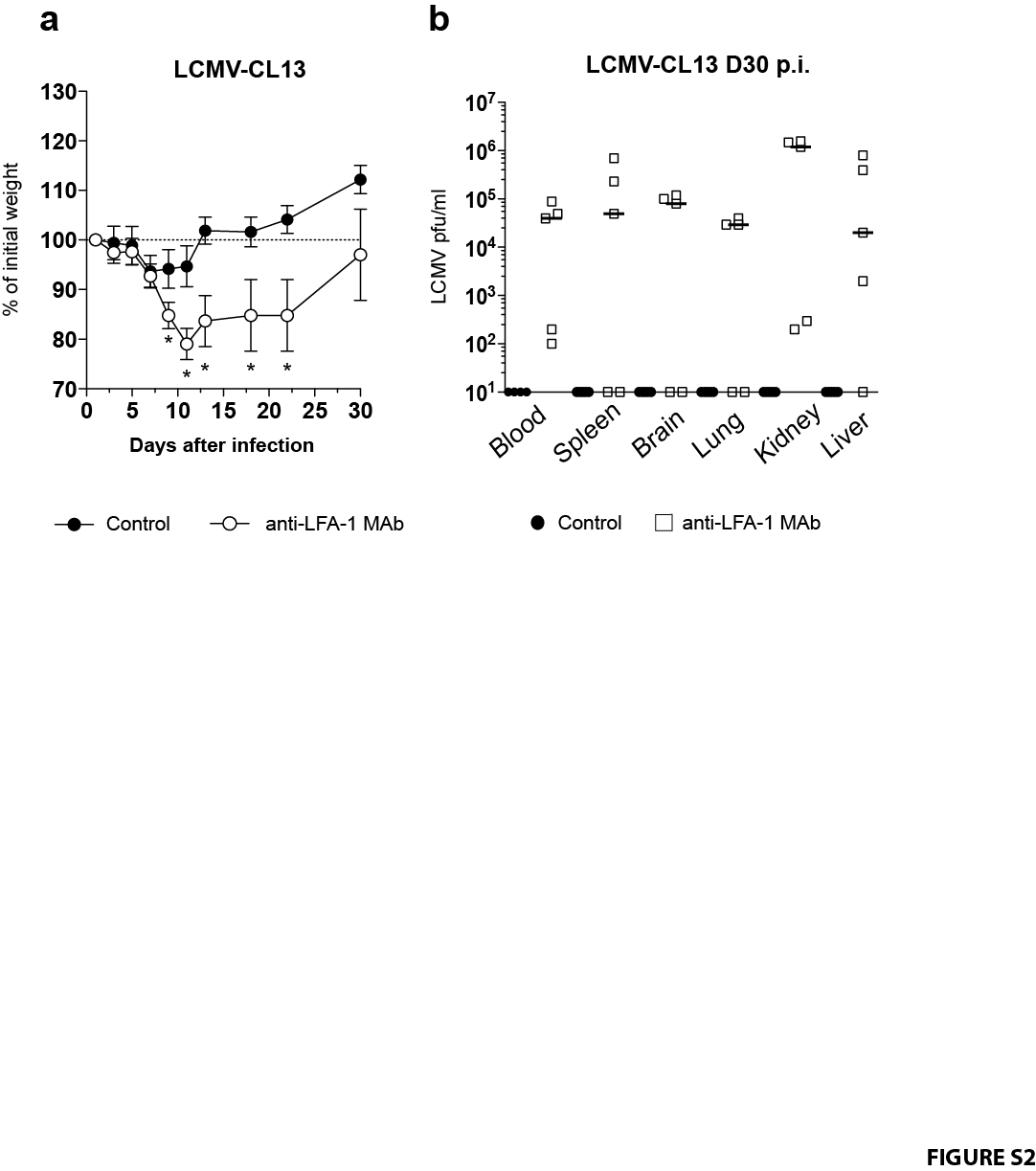
**

**Figure S2. Impact of anti-LFA-1 Mab antibody treatment on disease and control of LCMV-CL13 viremia.** (a) Monitoring of weight loss during LCMV-CL13 infection. (b) LCMV viral titers were determined by plaque assay in in several organs at day 30 post-infection for LCMV-CL13 infected mice. Detection limit 100pfu.

* p<0.05, ** p<0.001, *** p<0.0001. Error bars represent mean±SEM. Three independent experiments including 3 mice per group were performed with similar results.


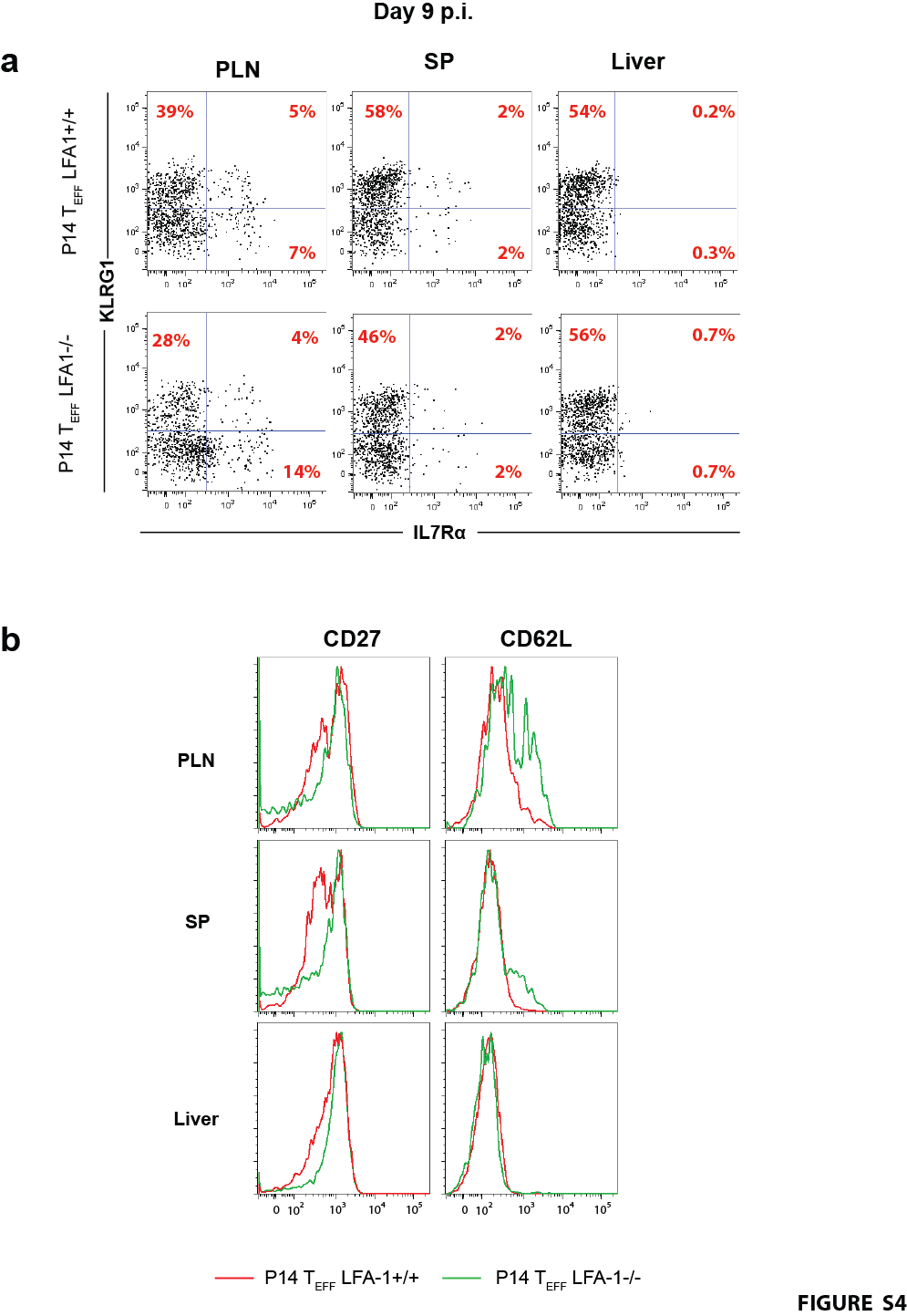


**Figure S3. Impact of LFA-1 deficiency in T_EFF_ differentiation after LCMV-CL13 infection.**

(a,b) Surface expression of KLRG1, IL7Rα, CD27 and CD62L was evaluated in the WT and LFA1^-/-^ P14 T_EFF_ from different organs at the day 9 p.i. with (5x10^4^pfu) LCMV-CL13.

Two independent experiments including 3 mice per group were performed with similar results.

**
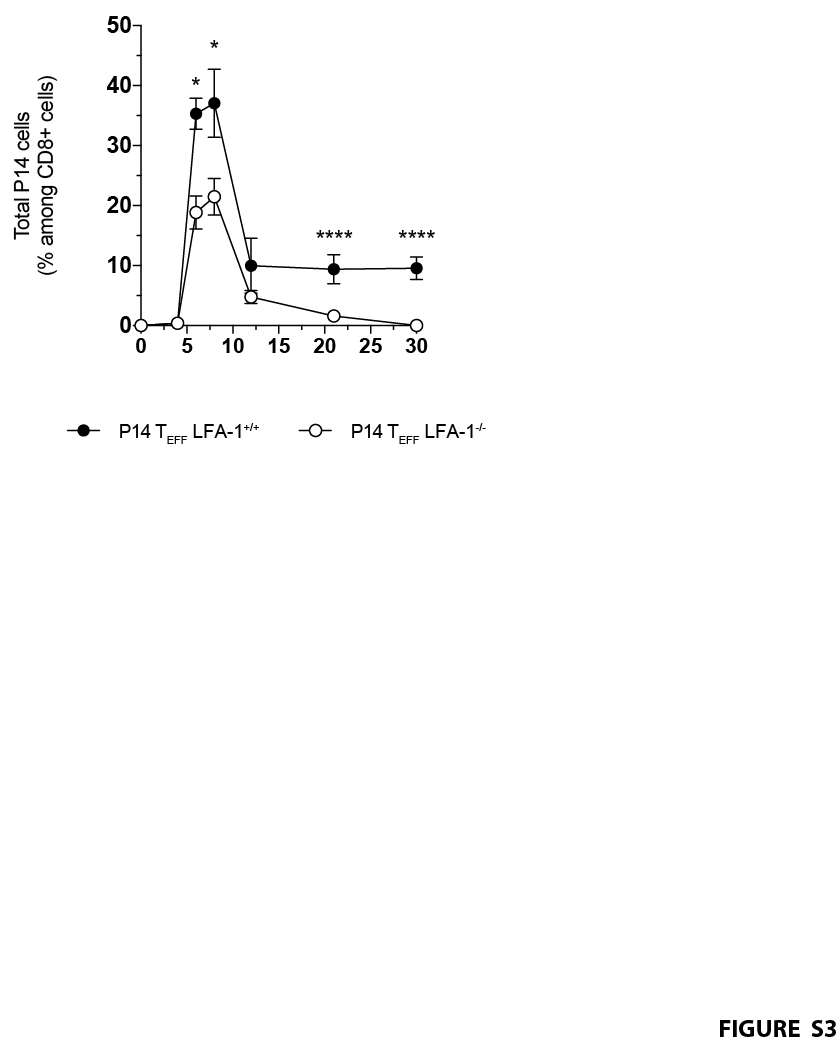
**

**Figure S4. Impact of LFA-1 on CD8 T cells burst size after LCMV-CL13 infection.**

A small number (10,000 cells) of naïve LFA-1^+/+^ CD45.1^+^ and LFA-1^-/-^ CD45.2^+^ P14 T_N_ cells were enriched by magnetic negative selection (>95% purity) and transferred into the separate WT CD45.2^+^ recipient, thus generating P14 chimeric mice. (c) 24 hours later, recipient mice were i.v. challenged with LCMV-CL13 (5x10^4^ pfu) that were able to clear infection with kinetics similar to non-chimeric mice. T_EFF_ burst size was quantified overtime in the blood of LCMV-CL13 infected mice by flow cytometry.

**
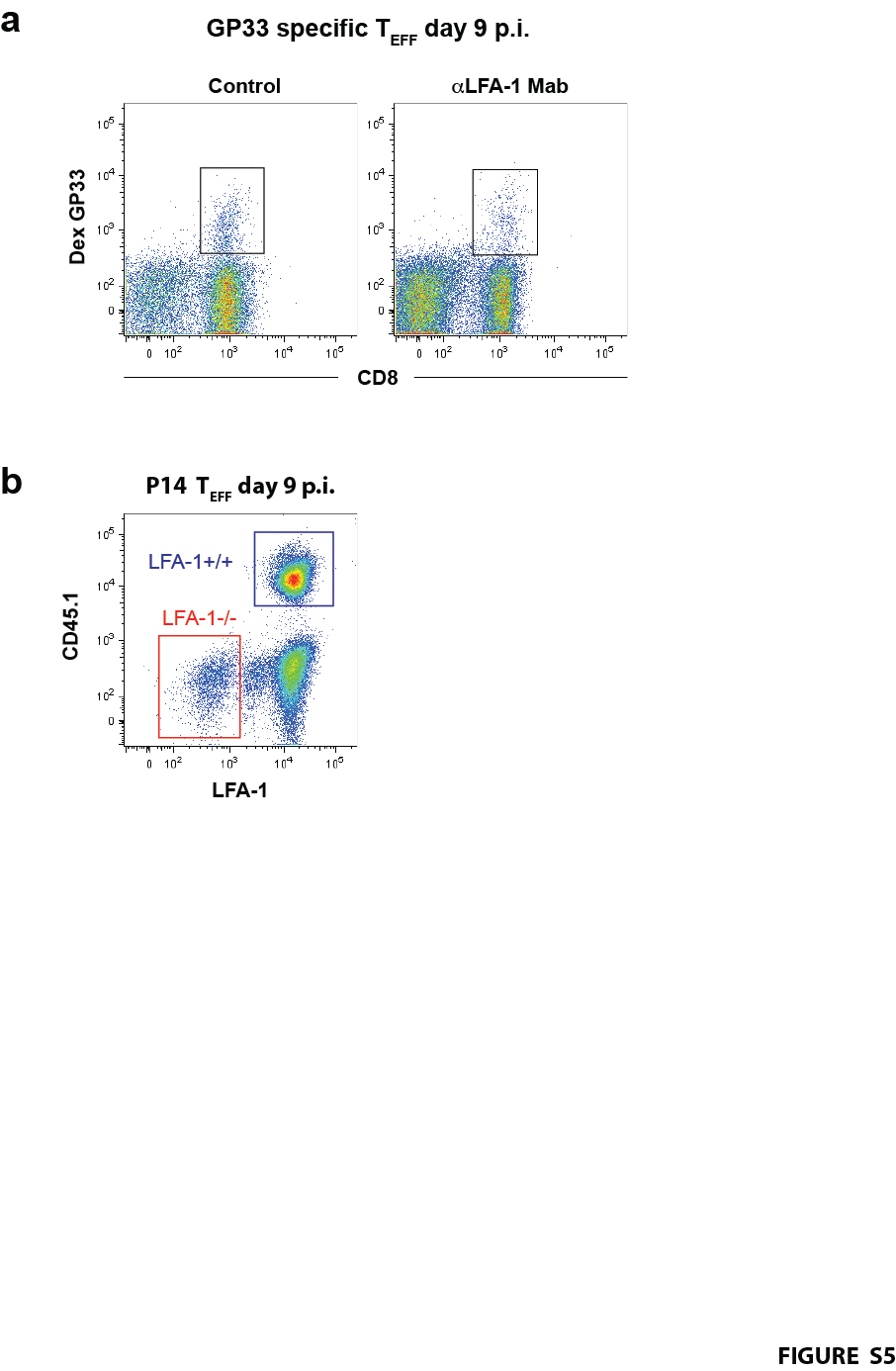
**

**Figure S5. Gating strategy for transcriptome analysis.** (a) DexGP-33 T_EFF_ were identified based on their surface expression of CD8 and binding to Dextramer GP33. (b) P14 T_EFF_ were identified based on their surface expression of LFA-1 and CD45.1 after gating on CD8+ lymphocytes.

**
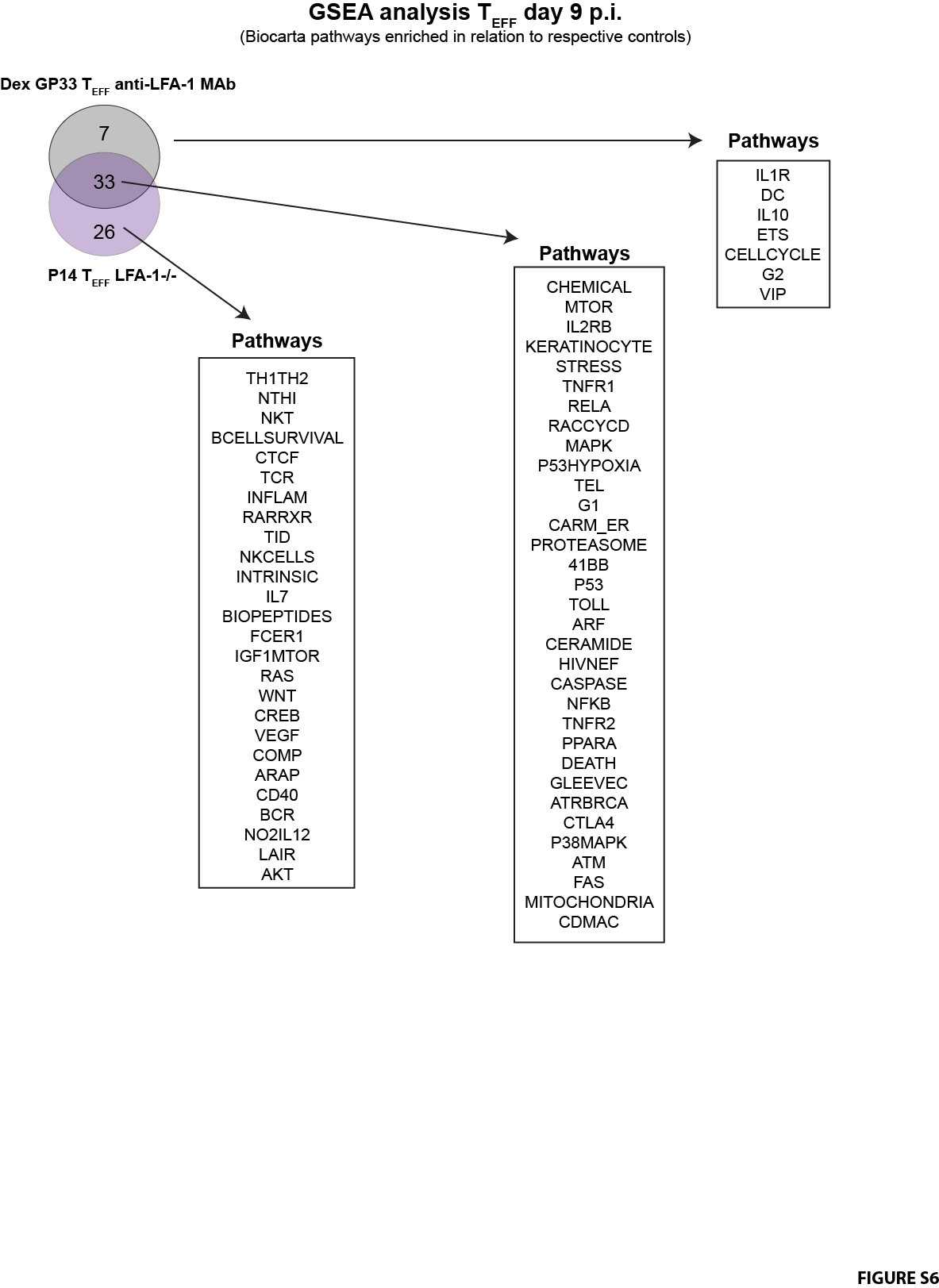
**

**Figure S6. GSEA analysis of P14 and DexGP-33 T_EFF_ generated during LCMV-CL13 infection.** (a) Overlap in the pathways identified by GSEA to be enriched in LFA1^-/-^ P14 T_EFF_ and DexGP-33 T_EFF_ from anti-LFA-1 Mab treated mice at day 9 p.i. when compared to LFA1^+/+^ P14 T_EFF_ and DexGP-33 T_EFF_ from control infected mice, respectively.

**
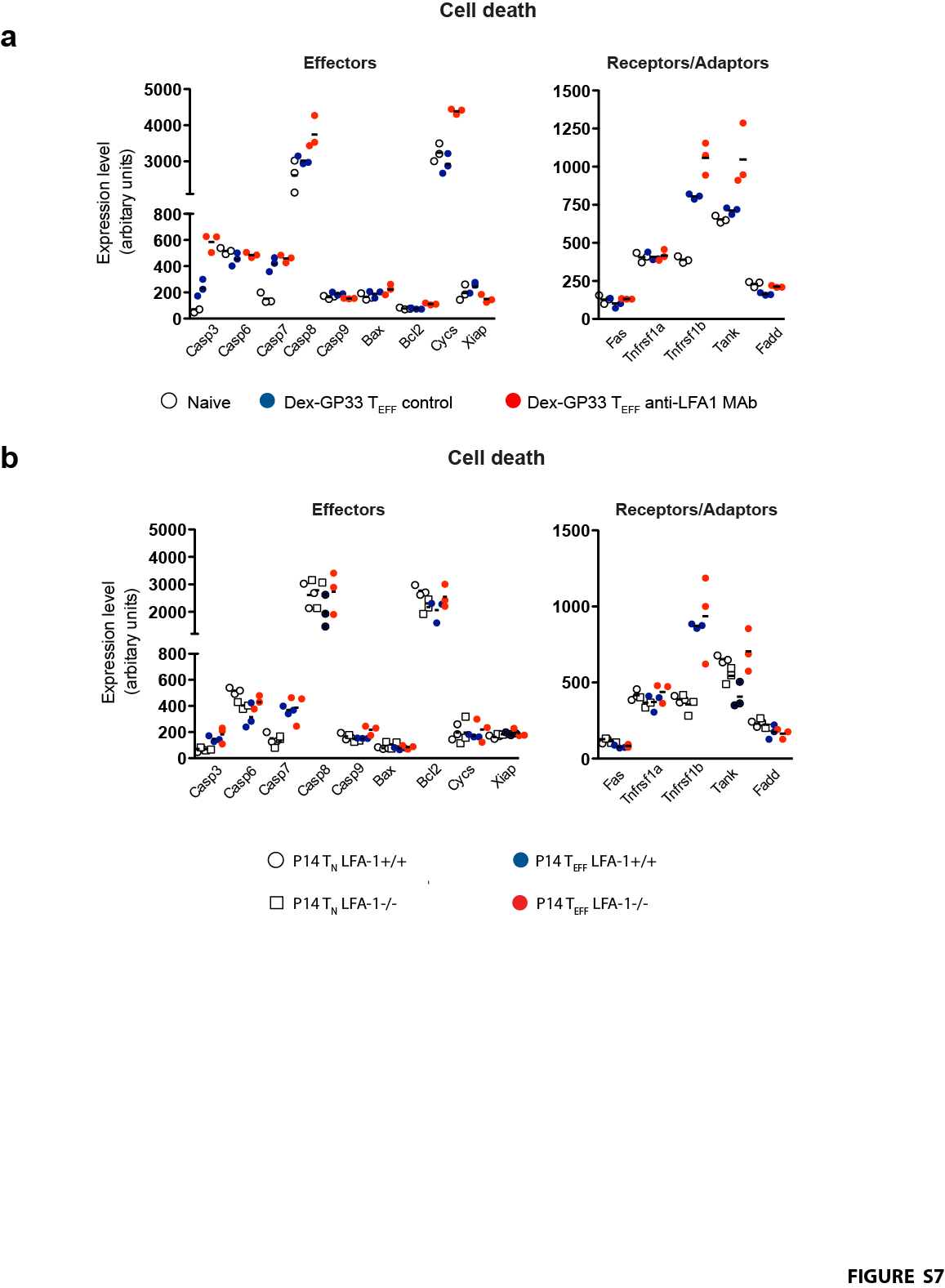
**

**Figure S7. Naïve P14, P14 and DexGP-33 T_EFF_ expression of a set of genes involved in cell death.**

(a) Relative expression levels of genes in Dex-GP-33 T_EFF_ from control and anti-LFA-1 Mab treated mice in comparison to P14 LFA1^+/+^ naïve cells. (b) Relative expression levels of genes in LFA1^+/+^ and LFA1^-/-^ P14 T_EFF_ in comparison to LFA1^+/+^ and LFA1^-/-^ P14 naïve cells.

**
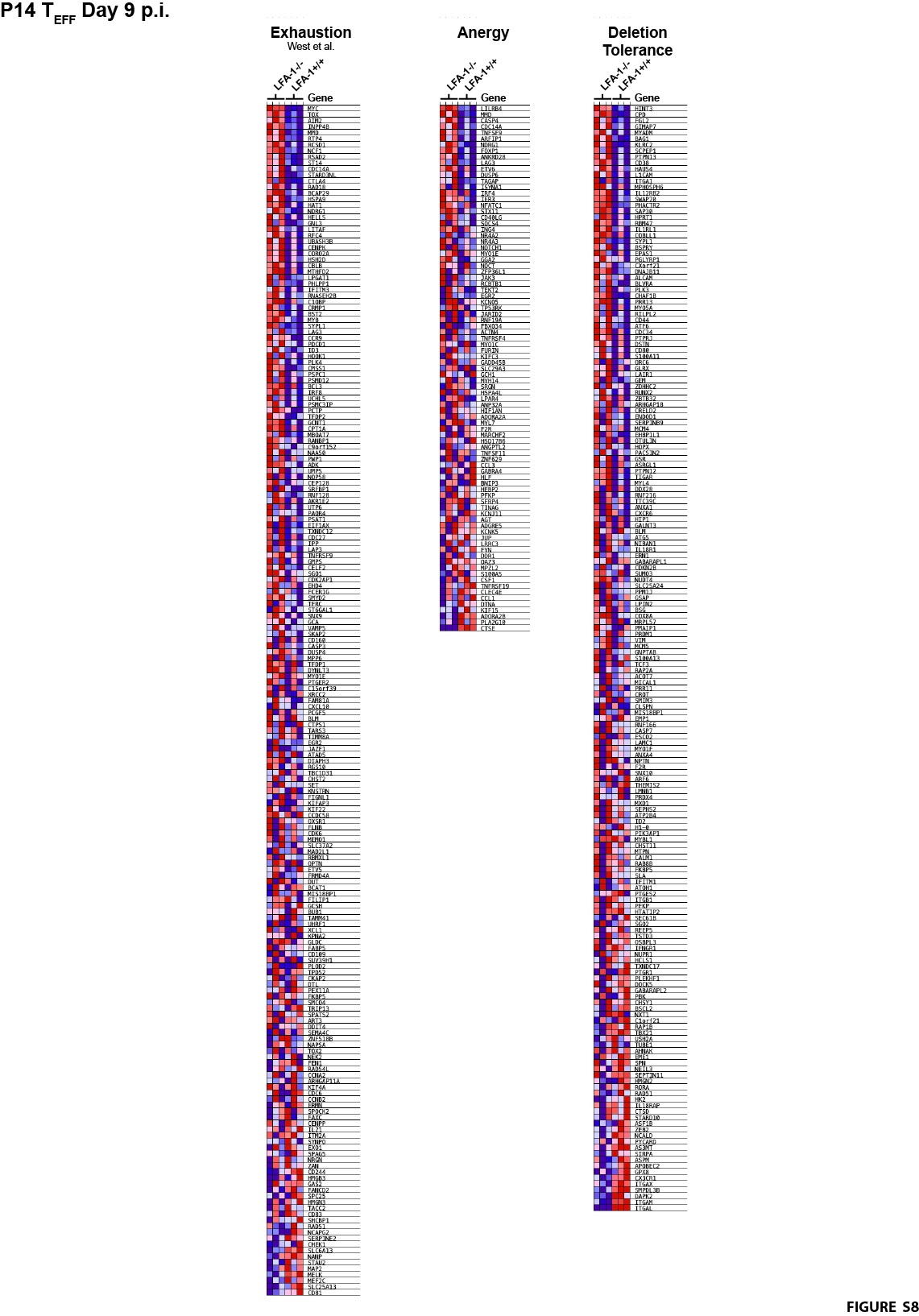
**

**Figure S8. Transcriptional signatures of exhaustion, anergy and deletion tolerance in T_EFF_ in P14 T_EFF_.** Geneset enrichment analysis (GSEA) on T cells exhaustion ^41^, anergy ^40^ and deletion tolerance ^39^signatures from MSigDB v7.1 (Broad institute) was performed on day 9 P14 T_EFF_ from LCMV-CL13 infection mice.

**
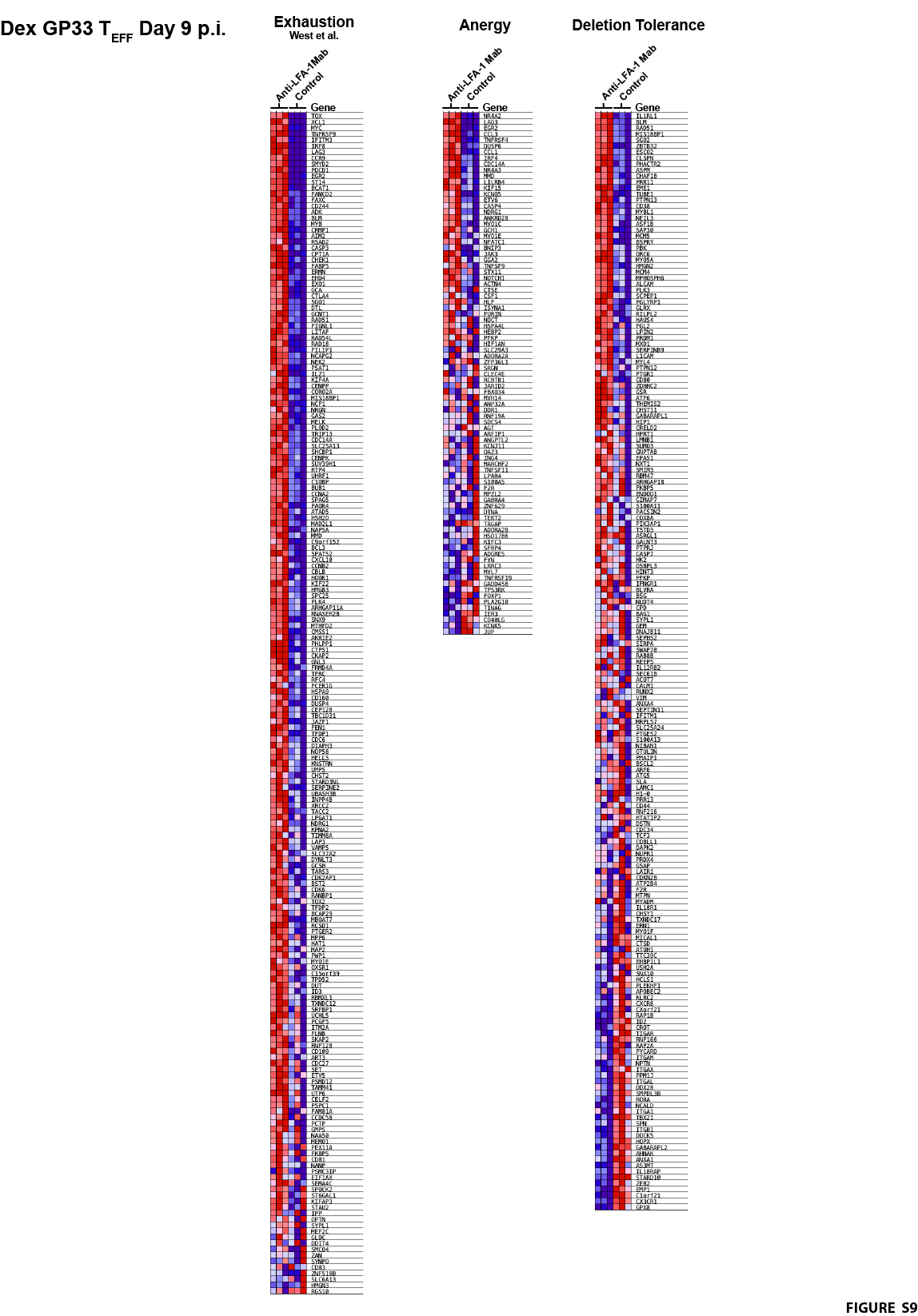
**

**Figure S9. Transcriptional signatures of exhaustion, anergy and deletion tolerance in Dex-GP33 specific.** Geneset enrichment analysis (GSEA) on T cells exhaustion ^41^, anergy ^40^ and deletion tolerance ^39^signatures from MSigDB v7.1 (Broad institute) was performed on day 9 Dex-GP33 T_EFF_ from LCLV-CL13 infection mice.

**
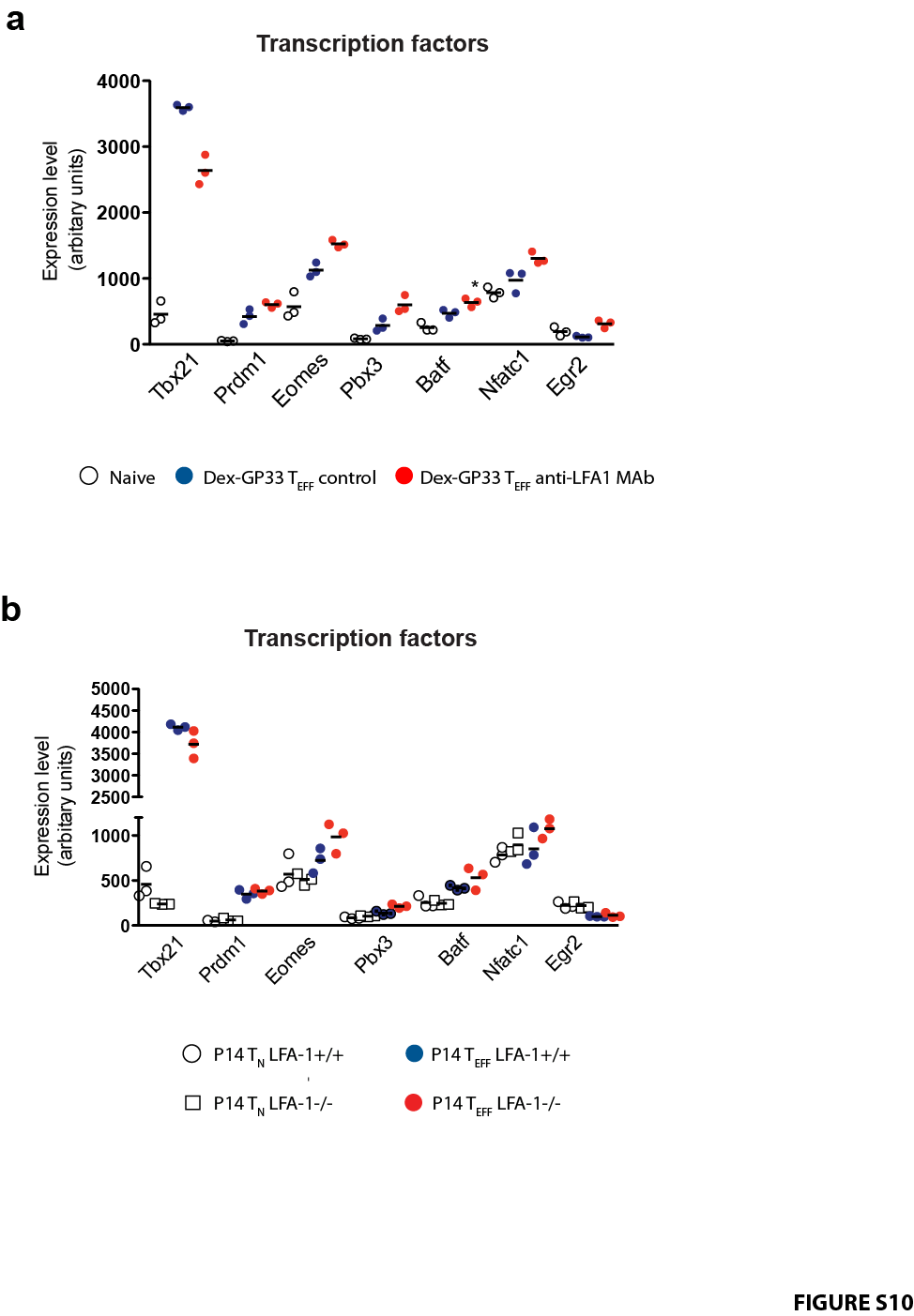
**

**Figure S10. Naïve P14, P14 and DexGP-33 T_EFF_ expression of a set of transcription factor genes involved in CD8 T cell function.** (a) Relative expression levels of genes in Dex-GP-33 T_EFF_ from control and anti-LFA-1 Mab treated mice in comparison to P14 LFA1^+/+^ naïve cells. (b) Relative expression levels of genes in LFA1^+/+^ and LFA1^-/-^ P14 T_EFF_ in comparison to LFA1^+/+^ and LFA1^-/-^ P14 naïve cells.

**
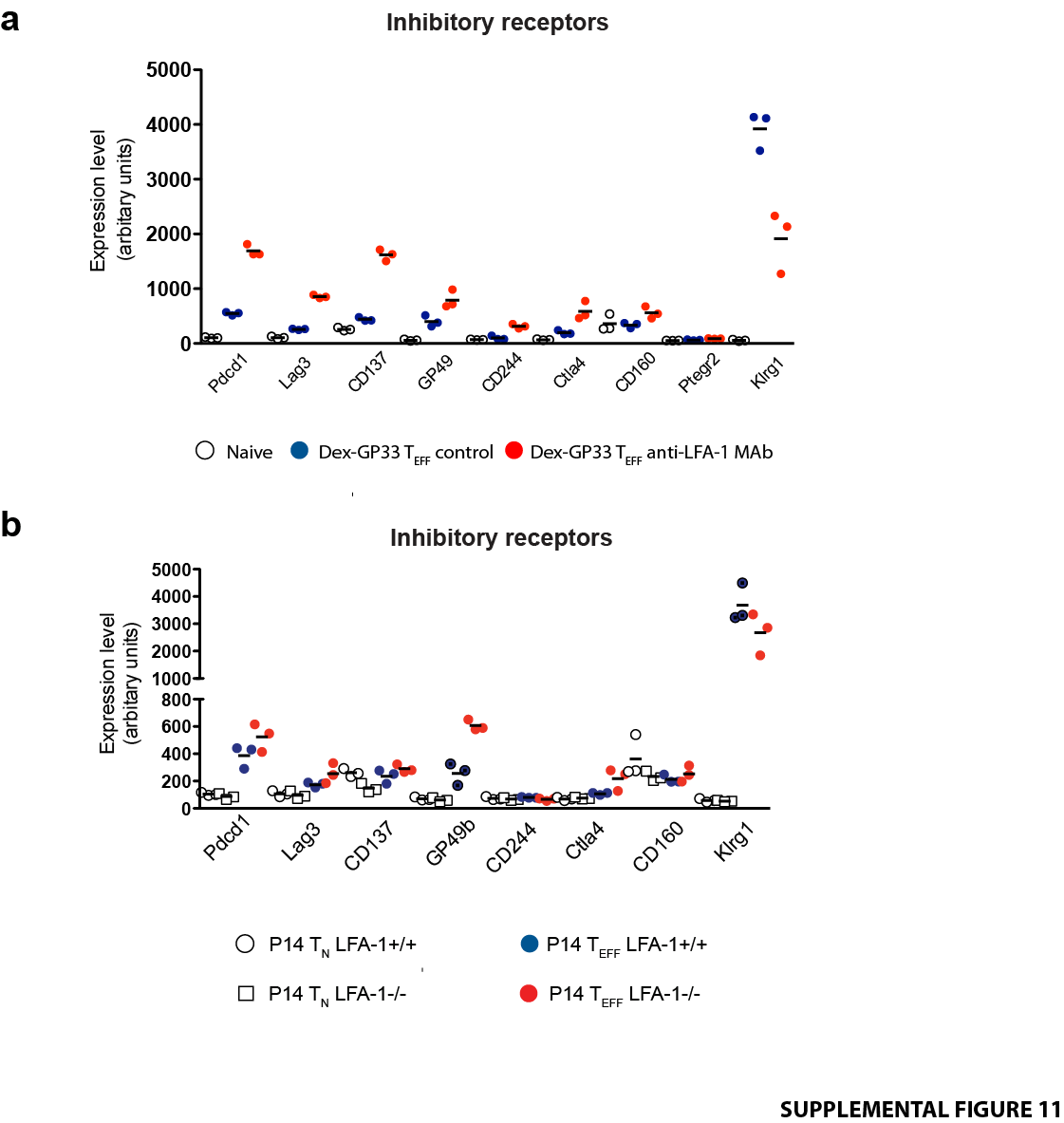
**

**Figure S11. Naïve P14, P14 and DexGP-33 T_EFF_ expression of a set of inhibitory receptor genes involved in CD8 T cell function.** (a) Relative expression levels of genes in Dex-GP-33 T_EFF_ from control and anti-LFA-1 Mab treated mice in comparison to P14 LFA1^+/+^ naïve cells. (b) Relative expression levels of genes in LFA1^+/+^ and LFA1^-/-^ P14 T_EFF_ in comparison to LFA1^+/+^ and LFA1^-/-^ P14 naïve cells.

**
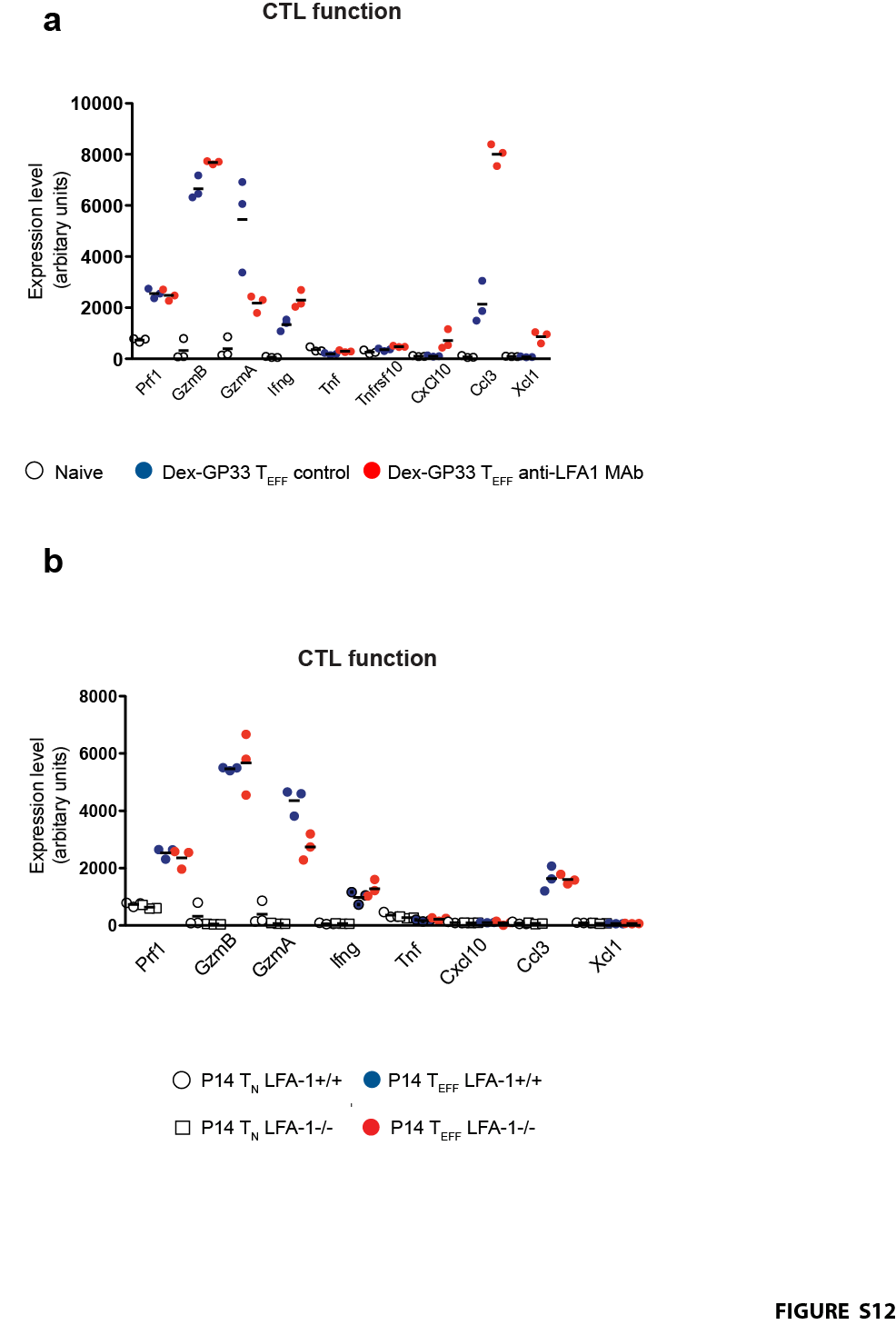
**

**Figure S12. Naïve P14, P14 and DexGP-33 T_EFF_ expression of a set of genes involved in CTL function.**

(a) Relative expression levels of genes in Dex-GP-33 T_EFF_ from control and anti-LFA-1 Mab treated mice in comparison to P14 LFA1^+/+^ naïve cells. (b) Relative expression levels of genes in LFA1^+/+^ and LFA1^-/-^ P14 T_EFF_ in comparison to LFA1^+/+^ and LFA1^-/-^ P14 naïve cells.

**Table S1. GSEA of BIOCARTA curated gene sets from the Molecular Signatures Database (MSigDB) specifically enriched on CD8 T cells upon anti-LFA-1 ab treatment.** GSEA analysis was used to identify cellular pathways significantly enriched in GP33+ specific CD8 T cells from 5x10^4^ pfu LCMV-CL13 infected mice treated with anti-LFA-1 Mab in comparison to those from PBS treated mice. Cut off was established as FDR <20% and p<0.05.

**Table S2. GSEA of BIOCARTA curated gene sets from the Molecular Signatures Database (MSigDB) specifically enriched on P14 LFA-1^-/-^ T cells after LCMV infection.** GSEA analysis was used to identify cellular pathways significantly enriched in day 9 p.i. LFA-1^-/-^ P14 T_EFF_ in comparison to LFA-1^+/+^ P14 T_EFF_ from 5x10^4^ pfu LCMV-CL13 infected mice. Cut off was established as FDR <20% and p<0.05.

**SUPPLEMENTAL REFERENCES**

1. von Andrian, U. H. & Mempel, T. R. Homing and cellular traffic in lymph nodes. *Nat. Rev. Immunol.* **3**, 867–878 (2003).

2. Link, A. *et al.* Fibroblastic reticular cells in lymph nodes regulate the homeostasis of naive T cells. *Nat. Immunol.* **8**, 1255–1265 (2007).

3. von Andrian, U. H. & Mackay, C. R. T-cell function and migration. Two sides of the same coin. *N. Engl. J. Med.* **343**, 1020–1034 (2000).

4. Park, E. J. *et al.* Distinct roles for LFA-1 affinity regulation during T-cell adhesion, diapedesis, and interstitial migration in lymph nodes. *Blood* **115**, 1572–1581 (2010).

5. Scholer, A., Hugues, S., Boissonnas, A., Fetler, L. & Amigorena, S. Intercellular adhesion molecule-1-dependent stable interactions between T cells and dendritic cells determine CD8+ T cell memory. *Immunity* **28**, 258–270 (2008).

6. Perez, O. D. *et al.* Leukocyte functional antigen 1 lowers T cell activation thresholds and signaling through cytohesin-1 and Jun-activating binding protein 1. *Nat. Immunol.* **4**, 1083–1092 (2003).

7. Lee, K.-H. *et al.* The immunological synapse balances T cell receptor signaling and degradation. *Science* **302**, 1218–1222 (2003).

8. Kaufman, Y. Lyt-2 negative and T cell growth factor independent cytotoxic T lymphocyte hybridomas. *Advances in Experimental Medicine and Biology* **146**, 435–446 (1982).

9. Kishimoto, T. K., Hollander, N., Roberts, T. M., Anderson, D. C. & Springer, T. A. Heterogeneous mutations in the beta subunit common to the LFA-1, Mac-1, and p150,95 glycoproteins cause leukocyte adhesion deficiency. *Cell* **50**, 193–202 (1987).

10. Schwab, N. *et al.* Fatal PML associated with efalizumab therapy: insights into integrin αLβ2 in JC virus control. *Neurology* **78**, 458–467; discussion 465 (2012).

11. Berlin-Rufenach, C. *et al.* Lymphocyte migration in lymphocyte function-associated antigen (LFA)-1- deficient mice. *J. Exp. Med.* **189**, 1467–1478 (1999).

12. Wohler, J., Bullard, D., Schoeb, T. & Barnum, S. LFA-1 is critical for regulatory T cell homeostasis and function. *Mol. Immunol.* **46**, 2424–2428 (2009).

13. Schmits, R. *et al.* LFA-1-deficient mice show normal CTL responses to virus but fail to reject immunogenic tumor. *J. Exp. Med.* **183**, 1415–1426 (1996).

14. Ghosh, S., Chackerian, A. A., Parker, C. M., Ballantyne, C. M. & Behar, S. M. The LFA-1 adhesion molecule is required for protective immunity during pulmonary Mycobacterium tuberculosis infection. *J. Immunol.* **176**, 4914–4922 (2006).

15. Prince, J. E. *et al.* The differential roles of LFA-1 and Mac-1 in host defense against systemic infection with Streptococcus pneumoniae. *J. Immunol.* **166**, 7362–7369 (2001).

16. Miyamoto, M. *et al.* Neutrophilia in LFA-1-deficient mice confers resistance to listeriosis: possible contribution of granulocyte-colony-stimulating factor and IL-17. *J. Immunol.* **170**, 5228–5234 (2003).

17. Ahmed, R., Salmi, A., Butler, L. D., Chiller, J. M. & Oldstone, M. B. Selection of genetic variants of lymphocytic choriomeningitis virus in spleens of persistently infected mice. Role in suppression of cytotoxic T lymphocyte response and viral persistence. *J Exp Med* **160**, 521–40 (1984).

18. Salvato, M., Borrow, P., Shimomaye, E. & Oldstone, M. B. Molecular basis of viral persistence: a single amino acid change in the glycoprotein of lymphocytic choriomeningitis virus is associated with suppression of the antiviral cytotoxic T-lymphocyte response and establishment of persistence. *J. Virol.* **65**, 1863–1869 (1991).

19. Matloubian, M., Kolhekar, S. R., Somasundaram, T. & Ahmed, R. Molecular determinants of macrophage tropism and viral persistence: importance of single amino acid changes in the polymerase and glycoprotein of lymphocytic choriomeningitis virus. *J. Virol.* **67**, 7340–7349 (1993).

20. Wherry, E. J., Blattman, J. N., Murali-Krishna, K., van der Most, R. & Ahmed, R. Viral persistence alters CD8 T-cell immunodominance and tissue distribution and results in distinct stages of functional impairment. *J. Virol.* **77**, 4911–4927 (2003).

21. Quddus, J., Kaplan, A. & Richardson, B. C. Anti-CD11a prevents deletion of self-reactive T cells in neonatal C57BR mice. *Immunology* **82**, 301–305 (1994).

22. Revilla, C., González, A. L., Conde, C., López-Hoyos, M. & Merino, J. Treatment with anti-LFA-1 alpha monoclonal antibody selectively interferes with the maturation of CD4- 8+ thymocytes. *Immunology* **90**, 550–556 (1997).

23. Peixoto, A. *et al.* CD8 single-cell gene coexpression reveals three different effector types present at distinct phases of the immune response. *J. Exp. Med.* **204**, 1193–1205 (2007).

24. Wong, P. & Pamer, E. G. Feedback regulation of pathogen-specific T cell priming. *Immunity* **18**, 499–511 (2003).

25. Wherry, E. J. & Kurachi, M. Molecular and cellular insights into T cell exhaustion. *Nat. Rev. Immunol.* **15**, 486–499 (2015).

26. Joshi, N. S. *et al.* Inflammation directs memory precursor and short-lived effector CD8(+) T cell fates via the graded expression of T-bet transcription factor. *Immunity* **27**, 281–95 (2007).

27. Pircher, H., Bürki, K., Lang, R., Hengartner, H. & Zinkernagel, R. M. Tolerance induction in double specific T-cell receptor transgenic mice varies with antigen. *Nature* **342**, 559–561 (1989).

28. Hamann, A. *et al.* Evidence for an accessory role of LFA-1 in lymphocyte-high endothelium interaction during homing. *J. Immunol.* **140**, 693–699 (1988).

29. Warnock, R. A., Askari, S., Butcher, E. C. & von Andrian, U. H. Molecular mechanisms of lymphocyte homing to peripheral lymph nodes. *J. Exp. Med.* **187**, 205–216 (1998).

30. Wirth, T. C., Pham, N.-L. L., Harty, J. T. & Badovinac, V. P. High initial frequency of TCR-transgenic CD8 T cells alters inflammation and pathogen clearance without affecting memory T cell function. *Mol. Immunol.* **47**, 71–78 (2009).

31. Murali-Krishna, K. *et al.* Counting antigen-specific CD8 T cells: a reevaluation of bystander activation during viral infection. *Immunity* **8**, 177–187 (1998).

32. Suresh, M., Singh, A. & Fischer, C. Role of tumor necrosis factor receptors in regulating CD8 T-cell responses during acute lymphocytic choriomeningitis virus infection. *J. Virol.* **79**, 202–213 (2005).

33. Wolfe, T. *et al.* Reduction of antiviral CD8 lymphocytes in vivo with dendritic cells expressing Fas ligand-increased survival of viral (lymphocytic choriomeningitis virus) central nervous system infection. *J. Immunol.* **169**, 4867–4872 (2002).

34. Kamperschroer, C. & Quinn, D. G. The role of proinflammatory cytokines in wasting disease during lymphocytic choriomeningitis virus infection. *J. Immunol.* **169**, 340–349 (2002).

35. Richter, K., Perriard, G. & Oxenius, A. Reversal of chronic to resolved infection by IL-10 blockade is LCMV strain dependent. *Eur. J. Immunol.* **43**, 649–654 (2013).

36. Varga, G. *et al.* LFA-1 contributes to signal I of T-cell activation and to the production of T(h)1 cytokines. *J. Invest. Dermatol.* **130**, 1005–1012 (2010).

37. Li, D., Molldrem, J. J. & Ma, Q. LFA-1 regulates CD8+ T cell activation via T cell receptor-mediated and LFA-1-mediated Erk1/2 signal pathways. *J. Biol. Chem.* **284**, 21001–21010 (2009).

38. Schietinger, A. & Greenberg, P. D. Tolerance and exhaustion: defining mechanisms of T cell dysfunction. *Trends Immunol.* **35**, 51–60 (2014).

39. Parish, I. A. *et al.* The molecular signature of CD8+ T cells undergoing deletional tolerance. *Blood* **113**, 4575–4585 (2009).

40. Safford, M. *et al.* Egr-2 and Egr-3 are negative regulators of T cell activation. *Nat. Immunol.* **6**, 472–480 (2005).

41. Wherry, E. J. *et al.* Molecular signature of CD8+ T cell exhaustion during chronic viral infection. *Immunity* **27**, 670–684 (2007).

42. West, E. E. *et al.* Tight regulation of memory CD8(+) T cells limits their effectiveness during sustained high viral load. *Immunity* **35**, 285–298 (2011).

43. Kao, C. *et al.* Transcription factor T-bet represses expression of the inhibitory receptor PD-1 and sustains virus-specific CD8+ T cell responses during chronic infection. *Nat Immunol* **12**, 663–71 (2011).

44. Paley, M. A. *et al.* Progenitor and terminal subsets of CD8+ T cells cooperate to contain chronic viral infection. *Science* **338**, 1220–1225 (2012).

45. Lebwohl, M. *et al.* A novel targeted T-cell modulator, efalizumab, for plaque psoriasis. *N. Engl. J. Med.* **349**, 2004–2013 (2003).

46. Carson, K. R. *et al.* Monoclonal antibody-associated progressive multifocal leucoencephalopathy in patients treated with rituximab, natalizumab, and efalizumab: a Review from the Research on Adverse Drug Events and Reports (RADAR) Project. *Lancet Oncol* **10**, 816–24 (2009).

47. Major, E. O., Frohman, E. & Douek, D. JC viremia in natalizumab-treated patients with multiple sclerosis. *N. Engl. J. Med.* **368**, 2240–2241 (2013).

48. Matloubian, M., Concepcion, R. J. & Ahmed, R. CD4+ T cells are required to sustain CD8+ cytotoxic T-cell responses during chronic viral infection. *J. Virol.* **68**, 8056–8063 (1994).

49. Anderson, D. C. & Springer, T. A. Leukocyte adhesion deficiency: An inherited defect in the Mac-1, LFA-1, and p150,95 glycoproteins. *Ann. Rev. of Med.* **38**, 175–194 (1987).

50. Li, Q. *et al.* Visualizing antigen-specific and infected cells in situ predicts outcomes in early viral infection. *Science* **323**, 1726–1729 (2009).

51. Gosert, R., Kardas, P., Major, E. O. & Hirsch, H. H. Rearranged JC virus noncoding control regions found in progressive multifocal leukoencephalopathy patient samples increase virus early gene expression and replication rate. *J Virol* **84**, 10448–56 (2010).

52. Bachmann, M. F. *et al.* Distinct roles for LFA-1 and CD28 during activation of naive T cells: adhesion versus costimulation. *Immunity* **7**, 549–57 (1997).

53. Henrickson, S. E. *et al.* T cell sensing of antigen dose governs interactive behavior with dendritic cells and sets a threshold for T cell activation. *Nat Immunol* **9**, 282–91 (2008).

54. Yamagata, T., Mathis, D. & Benoist, C. Self-reactivity in thymic double-positive cells commits cells to a CD8 alpha alpha lineage with characteristics of innate immune cells. *Nat. Immunol.* **5**, 597–605 (2004).

55. Irizarry, R. A. *et al.* Summaries of Affymetrix GeneChip probe level data. *Nucleic Acids Res* **31**, e15 (2003).

56. Reich, M. *et al.* GenePattern 2.0. *Nat. Genet.* **38**, 500–501 (2006).

57. Subramanian, A. *et al.* Gene set enrichment analysis: a knowledge-based approach for interpreting genome-wide expression profiles. *Proc. Natl. Acad. Sci. U.S.A.* **102**, 15545–15550 (2005).

58. Mootha, V. K. *et al.* PGC-1alpha-responsive genes involved in oxidative phosphorylation are coordinately downregulated in human diabetes. *Nat. Genet.* **34**, 267–273 (2003).
